# miR-24-3p secreted as extracellular vesicle cargo by cardiomyocytes inhibits fibrosis in human cardiac microtissues

**DOI:** 10.1093/cvr/cvae243

**Published:** 2024-11-11

**Authors:** Giorgia Senesi, Alessandra M Lodrini, Shafeeq Mohammed, Simone Mosole, Jesper Hjortnaes, Rogier J A Veltrop, Bela Kubat, Davide Ceresa, Sara Bolis, Andrea Raimondi, Tiziano Torre, Paolo Malatesta, Marie-José Goumans, Francesco Paneni, Giovanni G Camici, Lucio Barile, Carolina Balbi, Giuseppe Vassalli

**Affiliations:** Laboratories for Translational Research, Ente Ospedaliero Cantonale, Istituto Cardiocentro Ticino, Bellinzona, Switzerland; Faculty of Biomedical Sciences, Università della Svizzera italiana, Lugano, Switzerland; Department of Cell and Chemical Biology, Leiden University Medical Center, Leiden, The Netherlands; Center for Translational and Experimental Cardiology, University Hospital Zürich and University of Zürich, Zurich, Switzerland; Institute of Oncology Research (IOR), Oncology Institute of Southern Switzerland (IOSI), Switzerland; Department of Thoracic Surgery, Leiden University Medical Center, Leiden, The Netherlands; Department of Biochemistry, Cardiovascular Research Institute Maastricht, Maastricht University, The Netherlands; Department of Pathology, Maastricht University Medical Center, The Netherlands; Cellular Oncology Unit, IRCCS Ospedale Policlinico San Martino, Genova, Italy; Laboratories for Translational Research, Ente Ospedaliero Cantonale, Istituto Cardiocentro Ticino, Bellinzona, Switzerland; Institute of Biomedical Research, IRB, Bellinzona, Switzerland; Heart Surgery Unit, Cardiocentro Ticino Institute, EOC, Lugano, Switzerland; Cellular Oncology Unit, IRCCS Ospedale Policlinico San Martino, Genova, Italy; Department of Experimental Medicine (DIMES), Experimental Biology Unit, University of Genova, Genova, Italy; Department of Cell and Chemical Biology, Leiden University Medical Center, Leiden, The Netherlands; Center for Translational and Experimental Cardiology, University Hospital Zürich and University of Zürich, Zurich, Switzerland; Center for Molecular Cardiology, University of Zurich, Schlieren, Switzerland; Laboratories for Translational Research, Ente Ospedaliero Cantonale, Istituto Cardiocentro Ticino, Bellinzona, Switzerland; Faculty of Biomedical Sciences, Università della Svizzera italiana, Lugano, Switzerland; Faculty of Biomedical Sciences, Euler Institute, Università della Svizzera italiana, Lugano, Switzerland; Center for Molecular Cardiology, University of Zurich, Schlieren, Switzerland; Department of Medicine, Baden Cantonal Hospital, Baden, Switzerland; Laboratories for Translational Research, Ente Ospedaliero Cantonale, Istituto Cardiocentro Ticino, Bellinzona, Switzerland; Faculty of Biomedical Sciences, Università della Svizzera italiana, Lugano, Switzerland; Center for Molecular Cardiology, University of Zurich, Schlieren, Switzerland

**Keywords:** Cardiac fibrosis, Myocardial infarction, Extracellular vesicles, Cardiomyocytes, microRNA, Microtissues

## Abstract

**Aims:**

Cardiac fibrosis in response to injury leads to myocardial stiffness and heart failure. At the cellular level, fibrosis is triggered by the conversion of cardiac fibroblasts (CF) into extracellular matrix-producing myofibroblasts. miR-24-3p regulates this process in animal models. Here, we investigated whether miR-24-3p plays similar roles in human models.

**Methods and results:**

Gain- and loss-of-function experiments were performed using human induced pluripotent stem cell-derived cardiomyocytes (hCM) and primary hCF under normoxic or ischaemia-simulating conditions. hCM-derived extracellular vesicles (EVs) were added to hCF. Similar experiments were performed using three-dimensional human cardiac microtissues and *ex vivo* cultured human cardiac slices. hCF transfection with miR-24-3p mimic prevented TGFβ1-mediated induction of FURIN, CCND1, and SMAD4—miR-24-3p target genes participating in TGFβ1-dependent fibrogenesis—regulating hCF-to-myofibroblast conversion. hCM secreted miR-24-3p as EV cargo. hCM-derived EVs modulated hCF activation. Ischaemia-simulating conditions induced miR-24-3p depletion in hCM-EVs and microtissues. Similarly, hypoxia down-regulated miR-24-3p in cardiac slices. Analyses of clinical samples revealed decreased miR-24-3p levels in circulating EVs in patients with acute myocardial infarction (AMI), compared with healthy subjects. *Post-mortem* RNAScope analysis showed miR-24-3p down-regulation in myocardium from patients with AMI, compared with patients who died from non-cardiac diseases. Berberine, a plant-derived agent with miR-24-3p-stimulatory activity, increased miR-24-3p contents in hCM-EVs, down-regulated FURIN, CCND1, and SMAD4, and inhibited fibrosis in cardiac microtissues.

**Conclusion:**

These findings suggest that hCM may control hCF activation through miR-24-3p secreted as EV cargo. Ischaemia impairs this mechanism, favouring fibrosis.

## Graphical Abstract

**Graphical Abstract:**
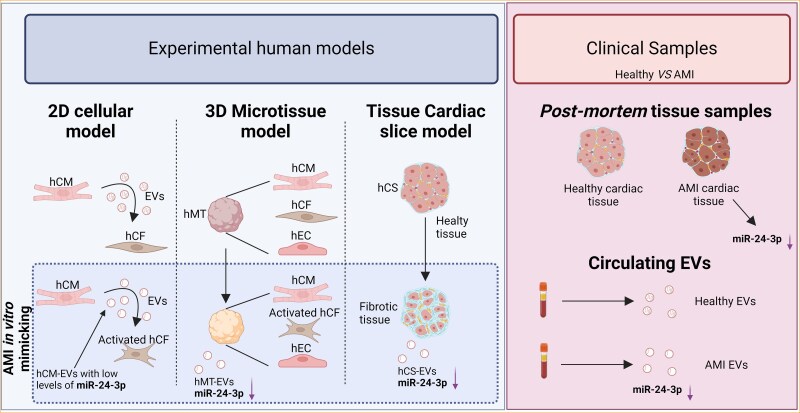

**Time of primary review: 15 days**

## 1. Introduction

Myocardial fibrosis, the expansion of the cardiac interstitium through deposition of extracellular matrix (ECM) proteins, plays central pathophysiological roles in various heart disease conditions, including acute myocardial infarction (MI), chronic ischaemia, heart failure with reduced or preserved ejection fraction, genetic cardiomyopathies, and diabetic heart disease. After MI, ECM deposition at sites remote from the infarct area results in cardiac stiffness, eventually leading to heart failure. A variety of fibrogenic growth factors [including transforming growth factor-β-1 (TGFβ1) and platelet-derived growth factors], pro-inflammatory cytokines, and neurohumoral pathways (including the angiotensin II/AT1 axis and aldosterone) can activate intracellular signalling cascades involved in fibrogenesis.^1–3^ At the cellular level, the activation of cardiac fibroblasts (CF) and their conversion into secretory and contractile cells—named myofibroblasts—which exhibit functional activities of fibroblasts and smooth muscle cells, plays a central role in the development of cardiac fibrosis. Myofibroblasts are the main source of structural ECM proteins in fibrotic hearts.^1^ In addition, these cells secrete matricellular proteins, e.g. periostin (POSTN).^4^ Therefore, pharmacological approaches that modulate myofibroblast activation in response to cardiac injury show therapeutic potential.

The molecular mechanisms that regulate the conversion of CF into activated myofibroblasts are incompletely understood. Available evidence supports significant roles for non-coding RNA transcripts including microRNAs (miRs) and long non-coding RNAs (lncRs)—which regulate gene expression at the post-transcriptional level in cells—in the regulation of fibroblast function.^5^ miRs target key fibrogenic cascades including TGFβ1/Smad, angiotensin II/MAPK, and RhoA/ROCK.^5^ A previous analysis of 194 different miRs, differentially expressed in inflamed hearts, revealed that 25.8% of the corresponding mimics modulated fibroblast function^6^ and that some of them also regulated cardiomyocyte (CM) and macrophage functions. miRs that promote cardiac fibrosis include miR-21, miR671-5p, and miR144-3p, among others.^7–9^ Conversely, miRs that negatively regulate cardiac fibrosis include miR-15, miR-29, miR-101, miR-1954, and miR-24-3p, among others.^10–13^ It also has been shown that CF can secrete miRs in the cardiac interstitium as extracellular vesicle (EV) cargo. The subsequent uptake of the released EVs by recipient cells can regulate their function.^14^ Multiple miRs species released from various donor cells as EV cargo have been implicated in the regulation of cardiac fibrosis.^15^

Focusing on miR-24-3p, Wang *et al*.^16^ previously showed that an increase in ECM protein expression is closely correlated with down-regulation of miR-24-3p in murine MI models and that up-regulation of miR-24-3p by synthetic miR-24-3p precursors reduces *in vitro* fibrosis and CF differentiation. Qu *et al*.^17^ subsequently showed that a lncRNA named myocardial infarction-associated transcript (MIAT) is up-regulated in murine MI models and is associated with miR-24-3p down-regulation and cardiac fibrosis. Knockdown of endogenous MIAT by siRNA restored the deregulated expression of miR-24-3p and reduced cardiac fibrosis. Zhang *et al*.^18^ described miR-24-3p down-regulation in transverse aortic constriction mice and in Ang Ⅱ-treated CF. miR-24-3p alleviated cardiac fibrosis by suppressing CF mitophagy via PHB2 down-regulation in these models. Lastly, Ma *et al*.^19^ demonstrated up-regulation of rhabdomyosarcoma 2-associated transcript (RMST) in association with cardiac fibrosis in murine and porcine MI models. RMST silencing *in vitro* inhibited CF proliferation and ECM production. miR-24-3p inhibition abolished RMST knockdown-mediated effects on CF fibrosis by regulating the lysis oxidase signalling pathway, whereas miR-24-3p agomir reproduced these effects. Collectively, these findings, using animal models, suggest that miR-24-3p may inhibit cardiac fibrosis. Some biological activities of miRs are species-specific, however, and data generated in animal models cannot be directly translated to human. Moreover, the cellular source of endogenous miR-24-3p in the heart is unclear.

Here, we aimed to investigate the role of miR-24-3p in cardiac fibrosis using human *in vitro* and *ex vivo* models, with a focus on the role of secreted EVs on this process.

## 2. Methods

A detailed Methods section is available as a supplementary information file.

## 3. Results

### 3.1 Bioinformatic analyses identify miR-24-3p as a prominently expressed miR in normal human hearts, and FURIN, CCND1, and SMAD4 as miR-24-3p target genes being up-regulated in CF from human MI hearts

**Figure 1:**
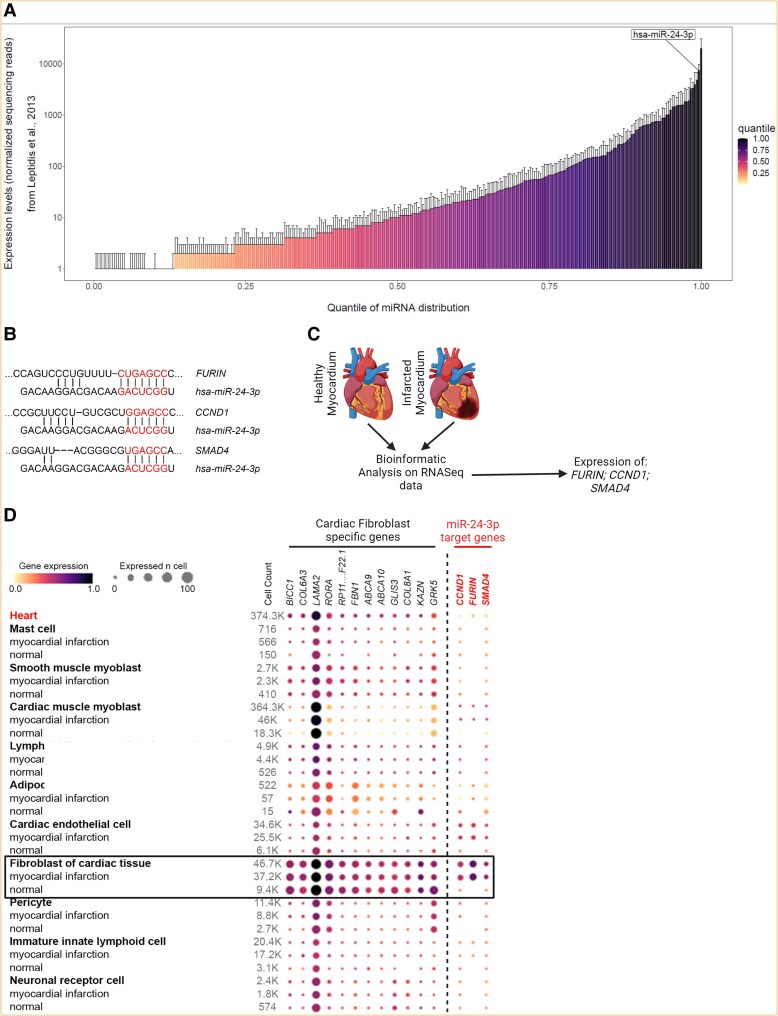
(*A*) Database analysis of miRs expression in normal human hearts. miR-24-3p ranked second among miRs showing highest expression levels. (*B*) Nucleotide sequence analysis predicting *FURIN*, *CCND1*, and *SMAD4* as target genes for miR-24-3p. (*C*) Cartoon depicting bioinformatic single-cell RNA-seq database analysis of heart tissue from healthy subjects and patients with MI. (*D*) *FURIN*, *CCND1*, and *SMAD4* were differentially expressed in hCF from patients with MI, whereas CF-specific genes were not.

Bioinformatic analyses on available human databases were carried out to gain initial insights into miR-24-3p expression and function in human hearts. The first bioinformatic analysis was performed on a small RNA sequencing database comparing >800 miRNAs expressed in the human heart.^20^ miR-24-3p was identified as the second most abundant miR species in hearts from healthy subjects (*Figure 1A*). Target genes for miR-24-3p include *FURIN*, *CCND1*, and *SMAD4*, as shown by sequence target analysis (*Figure 1B*). We then performed a computational analysis using data from an integrative high-resolution map of human cardiac remodelling after MI using single-cell gene expression, chromatin accessibility, and spatial transcriptomic profiling of multiple physiological zones at distinct time points in myocardium from patients after acute MI and healthy subjects^21^ (*Figure 1C*). No to low-level expression *of FURIN*, *CCND1*, and *SMAD4* was found in hCF obtained from normal hearts, whereas prominent expression of these genes was found in hCF from infarcted hearts (*Figure 1D*). The observation that miR-24-3p is prominently expressed in normal hearts, whereas its target genes are differentially expressed in hCF after MI supports a regulatory role for miR-24-3p in normal human hearts, which could be impaired after injury.

### 3.2 miR-24-3p regulates FURIN, CCND1, and SMAD4 expression in hCF

**Figure 2:**
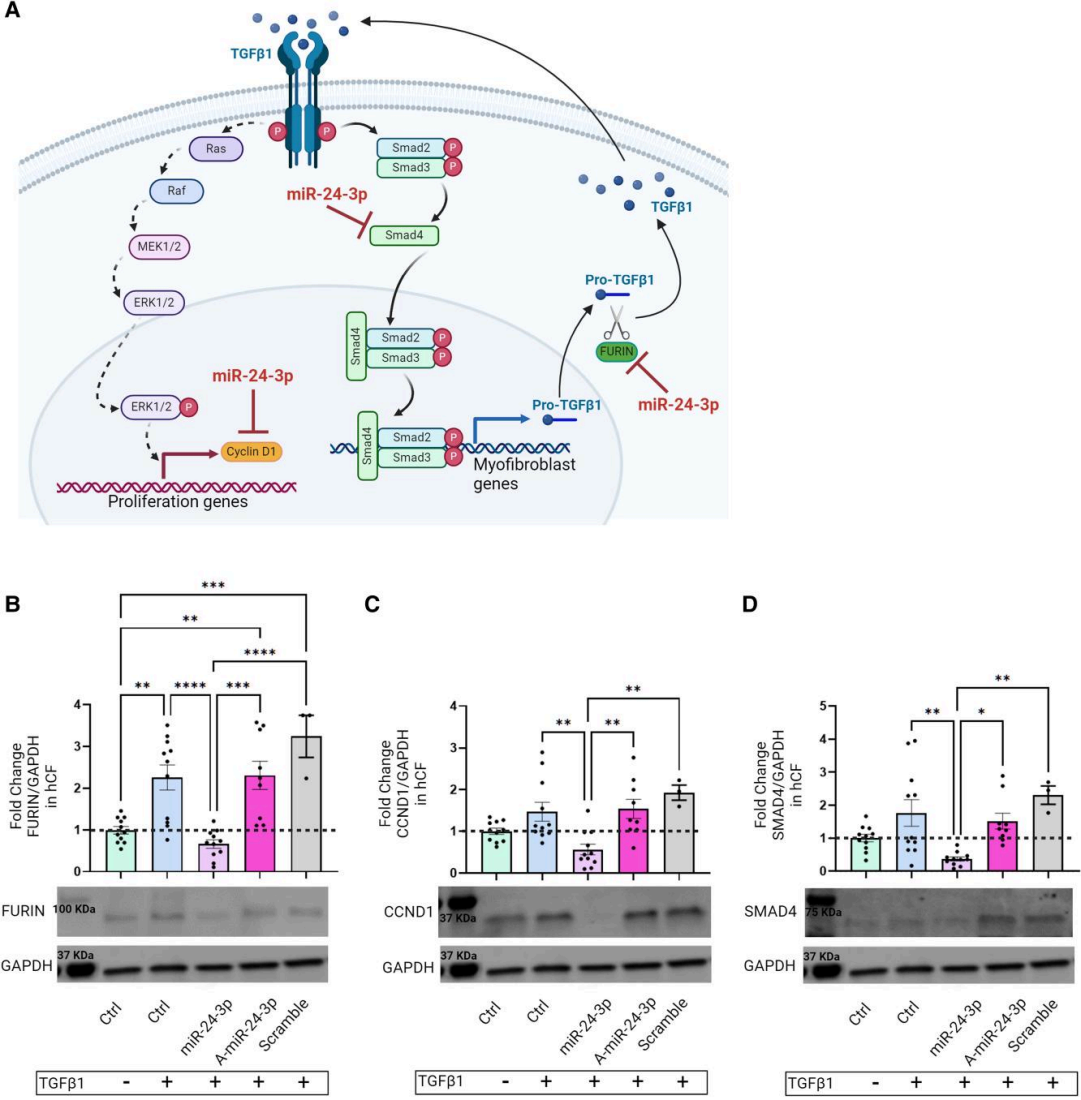
(*A*) Cartoon depicting TGFβ1-activated intracellular signalling pathways. Mechanisms of action of FURIN, CCND1, SMAD4, and miR-24-3p are indicated. (*B*) Western analysis of FURIN expression by TGFβ1-treated, miR-24-3p-transfected hCF at 72 h post-transfection. (*C*) Western analysis of CCND1 expression. (*D*) Western analysis of SMAD4 expression (data in panels *C*–*E* are fold-changes for miR-24-3p-transfected vs. naive hCF; anti-miR-24-3p, A-miR-24-3p; miR-scramble was used as a control miR; GAPDH was used as a loading control).

Available data suggest that FURIN, CCND1, and SMAD4 may be involved in TGFβ1-activated fibrogenic cascades^22^ (*Figure 2A*). Our STRING analysis data supported this assumption (see Supplementary material online, *Figure S1*). To assess whether miR-24-3p regulates FURIN, CCND1, and SMAD4 in hCF, we transfected these cells with a miR-24-3p mimic. Transfection efficacy was confirmed by real-time PCR (see Supplementary material online, *Figure S2A*). At the protein level, FURIN, CCND1, and SMAD4 down-regulation was observed at 72 h post-transfection (*Figure 2B–D* and Supplementary material online, *Figure S2B*). Interestingly, miR-24-3p-mediated FURIN, CCND1, and SMAD4 down-regulation was more pronounced in hCF stimulated with TGFβ1, when compared with untreated ones (see Supplementary material online, *Figure S2C–E*). Control transfection using miR-scramble showed no effect on protein regulation. Endoglin/CD105 and VIMENTIN—two genes that code for CF proteins but are not targets for miR-24-3p—were not down-regulated by this miR in hCF (see Supplementary material online, *Figure S2F–G*). Moreover, a miR-24-3p mimic decreased TGFβ1 secretion by activated hCF, as measured by ELISA (see Supplementary material online, *Figure S2H*), consistent with functional down-regulation of FURIN, which cleaves pro-TGFβ1 into TGFβ1. These findings indicate that miR-24-3p negatively regulates FURIN, CCND1, and SMAD4 expression in activated hCF.

### 3.3 miR-24-3p regulates hCF activation

**Figure 3:**
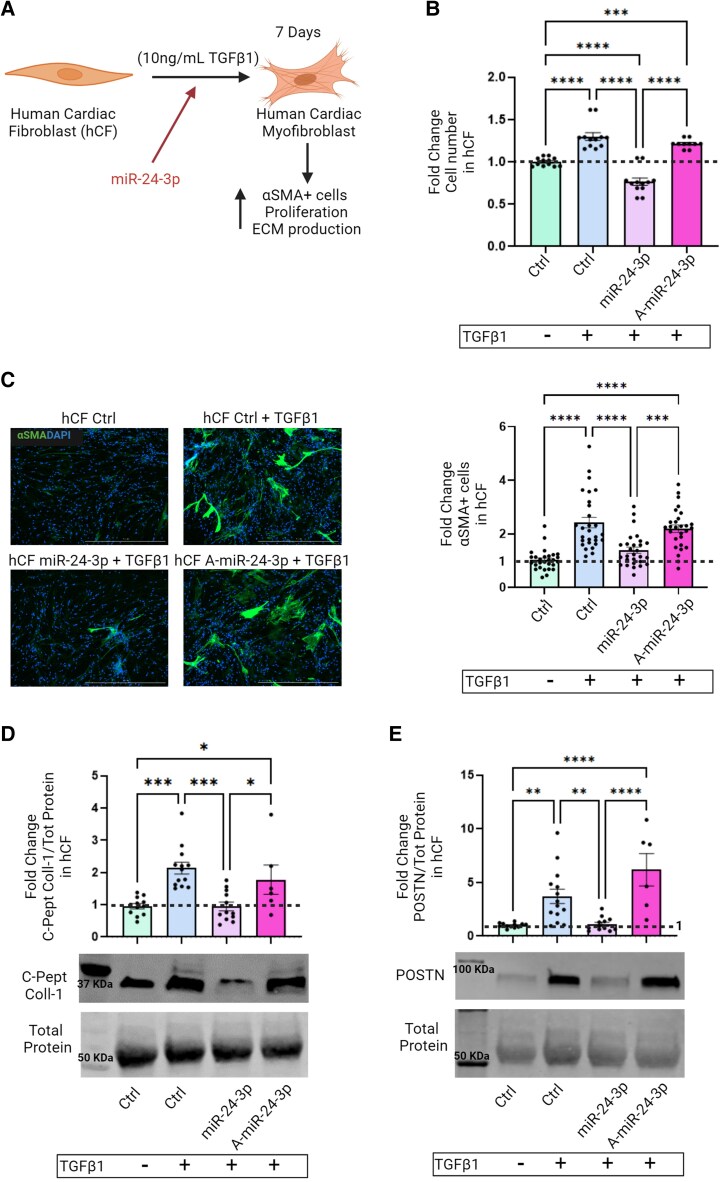
(*A*) Cartoon depicting miR-24-3p effects on hCF activation, proliferation, and ECM production. (*B*) hCF numbers at day 7 post-TGFβ1 treatment and transfection with a miR-24-3p mimic. (*C*) Left: Representative immunocytochemical images (αSMA, green; DAPI, blue; scale bar: 1000 µm). Right: Quantitative analysis of αSMA^+^ hCF. (*D*) Western analysis of collagen-1 C-peptide in conditioned media of TGFβ1-treated, miR-24-3p-transfected hCF. (*E*) Western analysis of POSTN. The miR-24-3p mimic reduced αSMA^+^ cell numbers, collagen-1 C-peptide, and POSTN expression (data are fold-changes for the indicated experimental conditions vs. naive hCF); anti-miR-24-3p (A-miR-24-3p). Total protein signal was used as a loading control.

We then assessed whether miR-24-3p-mediated FURIN, CCND1, and SMAD4 down-regulation was associated with functional changes in TGFβ1-treated hCF (*Figure 3A*). hCF transfected with a miR-24-3p mimic exhibited decreases in both cell proliferation (*Figure 3B* and Supplementary material online, *Figure S3A*) and their conversion into activated myofibroblasts, as evidenced by α-smooth muscle actin (αSMA) expression (*Figure 3C*Supplementary material online, *Figure S3B*) and ECM deposition, as demonstrated by c-peptide collagen-1 (*Figure 3D*Supplementary material online, *Figure S3C*) and POSTN quantification (*Figure 3E*Supplementary material online, Figure*S3D*). These results indicate that miR-24-3p inhibits the conversion of hCF into activated myofibroblasts *in vitro*.

### 3.4 miR-24-3p secreted by hCM as EV cargo inhibits hCF activation

**Figure 4:**
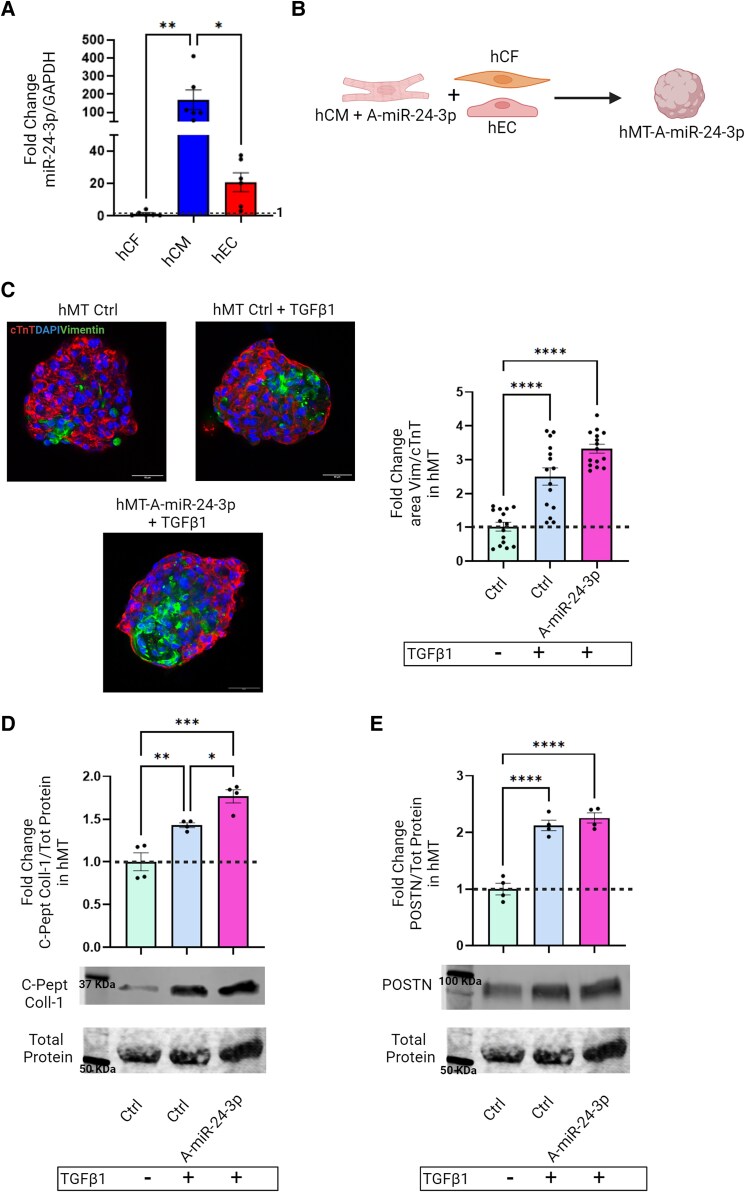
(*A*) Real-time PCR analysis of miR-24-3p expression in major cell types present in the heart (i.e. hCM, hCF, and hEC). (*B*) Cartoon depicting *in vitro* experiments using hMT composed of hCF, hEC, and hCM. The latter were transfected with anti-miR-24-3p (A-miR-24-3p). (*C*) Left: Representative immunofluorescence images (cTnT, red; Vimentin, green; DAPI, blue; scale bar: 50 µm). Right: Quantitative analysis of Vimentin^+^/cTnT^+^ area ratio in TGFβ1-treated hMT containing hCM transfected with A-miR-24-3p. (*D*) Western analysis of collagen-1 C-peptide in conditioned media of microtissues hMT containing either naïve hCM or hCM transfected with A-miR-24-3p. (*E*) Western analysis of POSTN (data in panels *C*–*E* are fold-changes for the indicated experimental conditions vs. hMT containing naive hCM). Total protein signal was used as a loading control.

Next, we measured *in vitro* expression of miR-24-3p in the three major cell types in the heart: CM, CF, and endothelial cells (EC). Human induced pluripotent stem cell (hiPS)-derived CMs (hCM) were used due to limited survival times of primary human CM in cell culture. hCM and primary hCF from ≥3 individual donors were used in each experiment. hCM, hEC, and hCF showed high, low, and undetectable levels of miR-24-3p expression, respectively (*Figure 4A*). These findings point to CM being the main cellular source of miR-24-3p in the heart. This raises the hypothesis that hCM functionally regulate hCF via a miR-24-3p-dependent mechanism. This hypothesis was tested using self-aggregating, three-dimensional (3D) tissue-like structures—named cardiac microtissues (hMT)—which are generated by co-culturing hCM, hCF, and hEC in the proportion of 1:0.2:0.2 (see Supplementary material online, *Figure S4A*).^23^ Three-cell-type cardiac hMT promote hCM maturation, as evidenced by sarcomere length and organization, contraction duration and amplitude, action potential profiles characterized by more hyperpolarized resting membrane potential, the presence of an action potential ‘notch’, and expression of post-natal sarcomere isoforms.^23^ hMT exhibited spontaneous beating activity at the time experiments were carried out. Moreover, the protocol allowed replacement of one of the three heart cell types in hMT with a modified variant. Here, hCM were transfected with anti-miR-24-3p prior to their co-incubation with hCF and hEC for hMT generation (*Figure 4B*). These hMT exhibited increases in vimentin^+^/cardiac troponin T^+^ (cTnT^+^) area ratios (*Figure 4C*) and c-peptide collagen-I expression (*Figure 4D*), when compared with hMT containing naïve hCM; POSTN expression was unchanged (*Figure 4E*). hCM transfection with a miR-24-3p mimic reversed TGFβ1-mediated FURIN, CCND1, and SMAD4 up-regulation in hMT (see Supplementary material online, *Figure S4B–D*). These results indicate that miR-24-3p expression by hCM inhibits hCF activation.

**Figure 5:**
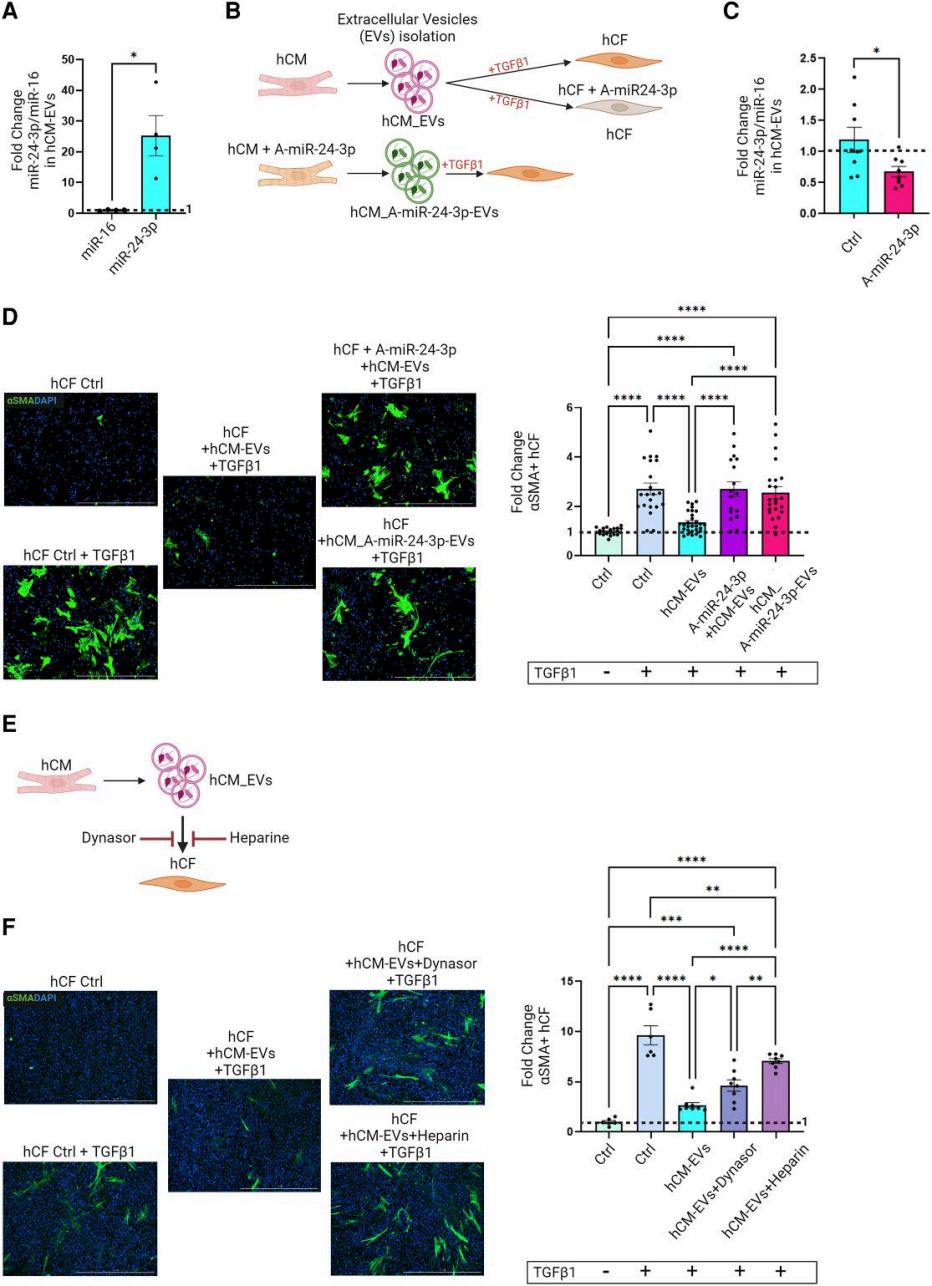
(*A*) ddPCR analysis of miR-24-3p in isolated hCM-EVs (miR-16, an abundant and stable miR, normally used as housekeeping in EVs, was measured for comparison). (*B*) Study design of loss-of-function experiments using hCM-EVs. Upper panel: EVs from naïve CM were added to either naïve hCF or hCF transfected with anti-miR-24-3p (A-miR-24-3p). Lower panel: EVs from hCM transfected with A-miR-24-3p were added to naïve hCF. (*C*) Real-time PCR analysis of miR-24-3p/miR-16 copy number ratios in EVs from hCM transfected with A-miR-24-3p (hCM_A-miR-24-3p-EVs) and EVs from naïve hCM (Ctrl). (*D*) Left: Representative immunocytochemical images (αSMA, green; DAPI, blue; scale bar: 1000 µm). Right: Quantitative analysis of αSMA-positive hCF after incubation with EVs from naive hCM (hCM-EVs), A-miR-24-3p in combination with hCM-EVs (A-miR-24-3p+ hCM-EVs), and EVs from hCM transfected with A-miR-24-3p (hCM_A-miR-24-3p-EVs). (*E*) Study design of experiments using chemical inhibitors of cellular EVs uptake (dynasore and heparin). (*F*) Left: representative immunocytochemical images (αSMA, green; DAPI, blue; scale bar: 1000 µm). Right: Quantitative analysis of αSMA^+^ cells after TGFβ1 treatment and incubation with hCM-EVs, with or without dynasore or heparin (data in *Figure 1D and F* are fold-changes for the indicated experimental conditions vs. naïve hCF).

Because EVs are key mediators of intercellular communication, we asked whether secreted EVs are involved in miR-24-3p-mediated hCF inhibition. EVs were isolated from hCM conditioned medium according to MISEV Guidelines^24^ by a two-step protocol combining size exclusion chromatography and ultracentrifugation (see Supplementary material online, *Figure S5A*). Using this method, a population of small hCM-derived EVs (hCM-EVs) was isolated, as demonstrated by nanotracking analysis (NTA; Supplementary material online, *Figure S5B*) and Western analysis of EV markers (CD63, CD81, TSG101, syntenin-1). GRP94 was used as a marker of intracytoplasmic proteins (see Supplementary material online, *Figure S5C*). Transmission electron microscopy (TEM) images of hCM-EVs are shown in Supplementary material online, *Figure S5D*. Flow cytometry analysis of EV surface markers (CD9, CD63, and CD81), the CM marker SIRPA/CD172a,^25^ and endoglin/CD105 (expressed in iPS-derived hCM^26^) is shown in Supplementary material online, *Figure S5E*. Digital PCR analysis of isolated hCM-EVs showed high miR-24-3p contents (*Figure 5A*); miR-16, a miR known for its abundance and stability,^27^ was measured as a housekeeping miR. To investigate the role of hCM-EVs in the functional regulation of hCF, hCM were transfected with anti-miR-24-3p (*Figure 5B*), resulting in a decrease in miR-24-3p levels in EV cargoes by approximately half (*Figure 5C*). EVs from naïve hCM did not affect αSMA^+^ cell numbers in TGFβ1-stimulated hCF that had been transfected with anti-miR-24-3p. Similar results were obtained when naïve hCF were incubated with EVs from hCM transfected with anti-miR-24-3p (hCM_A-miR-24-3p-EVs; *Figure 5D*). These results indicate that hCM-EV-mediated inhibition of hCF activation relies upon their miR-24-3p cargo levels.

To further address the role of hCM-EVs on hCF function, we used GW4869, a chemical inhibitor of EVs release^28^ (see Supplementary material online, *Figure S6A*). EVs secretion was assessed by CD63 dot blot (see Supplementary material online, *Figure S6B*). EVs preparations from GW4869-treated hCM failed to inhibit TGFβ1-mediated CF activation, when compared with volume-matched hCM-EVs preparations produced in the absence of GW4869 (see Supplementary material online, *Figure S6C*). In separate experiments, hCF were treated with dynasore or heparin, two chemical inhibitors of EVs cellular uptake,^29^ prior to their exposure to hCM-EVs (*Figure 5E*). These agents attenuated hCM-EVs-mediated inhibition of TGFβ1-induced increases in αSMA^+^ cells (*Figure 5F* and Supplementary material online, *Figure S6D*). EVs uptake was visualized by using DiR-labelled EVs (see Supplementary material online, *Figure S6E*). Collectively, these results indicate that hCM attenuate CF activation through miR-24-3p transfer as EV cargo.

### 3.5 Ischaemia-simulating conditions reduce miR-24-3p expression in hCM and promote hCF activation in hMT and *ex vivo* cultured cardiac slices

**Figure 6:**
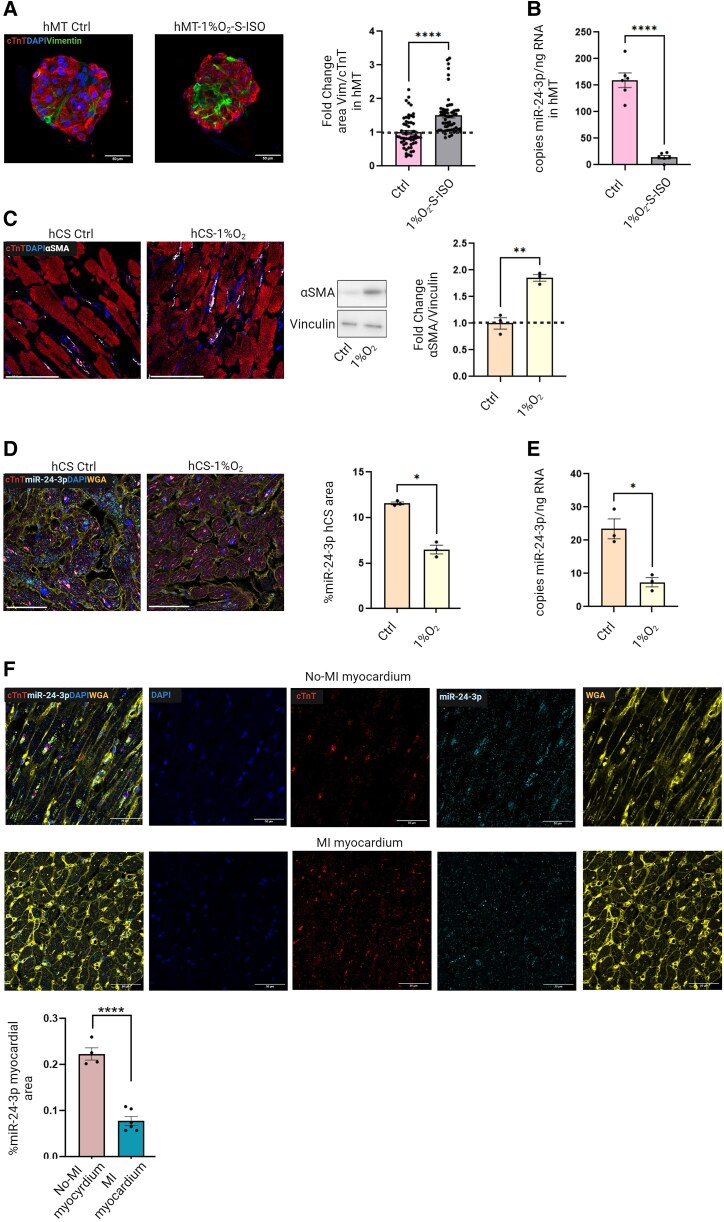
(*A*) Effects of ischaemia-like conditions on miR-24-3p expression and fibrosis in human cardiac microtissues. Left: Immunofluorescence staining of microtissues cultured under either normoxic (hMT) or ischaemia-like conditions [i.e. hypoxia (1% O_2_)/serum starvation (S)/500 µM ISO; (hMT-1%O_2_-S-ISO)] for vimentin (Vimentin, green; cTnT, red; DAPI, blue; scale bar: 50 µm). Right: Quantitative analysis of vimentin^+^/cTnT^+^ area ratios. (*B*) ddPCR analysis of miR-24-3p copy numbers/ng RNA in EVs from normoxic hMT and from hMT-1%O_2_-S-ISO. (*C*) Effects of hypoxia (1% O_2_) on miR-24-3p expression and hCF activation in *ex vivo* cultured hCS. Left: Immunofluorescence staining of hCS cultured under either normoxic or hypoxic conditions (hCS-1%O_2_; αSMA, white; cTnT, red; DAPI, blue; scale bar: 50 µm). Right: Western analysis for αSMA and vinculin. Quantitative analysis of αSMA expression (normalized for vinculin expression). (*D*) Left: Representative images of RNAScope staining for miR-24-3p in normoxic hCS and hypoxic hCS-1%O_2_ [cTnT mRNA, red; miR-24-3p, cyan; DAPI, blue; wheat germ agglutinin (WGA), yellow; scale bar: 50 µm]. Right: Quantitative analysis of miR-24-3p-stained area (%). (*E*) ddPCR analysis of miR-24-3p expression in normoxic hCS and hypoxic hCS-1%O_2_. (*F*) Representative images of RNAScope staining for miR-24-3p in *post-mortem* heart tissues from patients with AMI and patients who died from other diseases in the absence of overt heart disease (non-MI; cTnT mRNA, red; miR-24-3p, cyan; DAPI, blue; WGA, yellow; scale bar: 50 µm). Quantitative analysis of miR-24-3p-stained area (%).

Because TGFβ1 is rapidly and abundantly secreted in the myocardium after acute MI,^30^ recombinant-TGFβ1 can be used to mimic, to some extent, CF activation *in vitro*. Other models involving hypoxia and starvation may more closely mimic ischaemia. Accordingly, we cultured cardiac hMT under hypoxic conditions (1% O_2_) and serum starvation (S), in combination with isoproterenol (ISO; 500 µM)^31^ during 7 days (hMT-1%O_2_-S-ISO). Compared with normoxic hMT, hMT-1%O_2_-S-ISO exhibited increased Vimentin^+^/cTnT^+^ area ratios (*Figure 6A*), markedly decreased miR-24-3p expression levels (*Figure 6B*), and increased numbers of hCM showing evidence of cellular stress, with no changes in total hCM (see Supplementary material online, *Figure S7A and B*).

Finally, we used *ex vivo* cultured cardiac slices obtained from surgical waste material from patients who underwent valve replacement surgery or Morrow myectomy, as described previously.^32^ The thinness of these slices (300 µm) allows for direct absorption of oxygen and nutrients from the culture media without the need for external perfusion, resulting in prolonged viability and functionality in culture, which offers a unique research model for studying adult hCM over extended periods of time. For practical reasons, we used a previously validated hypoxia protocol in this advanced model (whereas the effects of serum starvation and ISO treatment on this model had not been assessed). After 7 days of hypoxia, cardiac slices exhibited increased αSMA expression (*Figure 6C*) and decreased miR-24-3p expression, measured by both RNAScope analysis (*Figure 6D*) and ddPCR (*Figure 6E*), when compared with cardiac slices cultured under normoxic conditions. Hypoxic cardiac slices exhibited no significant changes in hCM death but increased levels of hCM showing evidence of cellular stress, e.g. increased lipofuscin staining (see Supplementary material online, *Figure S7C and D*).

### 3.6 *Post-mortem* analysis of human cardiac tissues

We also performed a *post-mortem* analysis of human cardiac tissues from patients with MI and age-matched patients who had died from other causes not associated with primary cardiac injury (see Supplementary material online, *Figure S8*). Tissue samples were obtained within 48 h (mean: 29 h) of death (see Methods section). RNAScope analysis indicated decreased miR-24-3p expression levels in hearts from patients with MI, when compared with those who had died from extracardiac causes (*Figure 6F*). Collectively, these results indicate that both *ex vivo* hypoxic cardiac injury and *in vivo* ischaemic injury, as assessed at *post-mortem* examination, are associated with miR-24-3p down-regulation, which results in enhanced CF activation.

### 3.7 Ischaemia-simulating conditions reduce miR-24-3p secretion as EVs cargo in hCM, hMT, and *ex vivo* cultured cardiac slices

**Figure 7:**
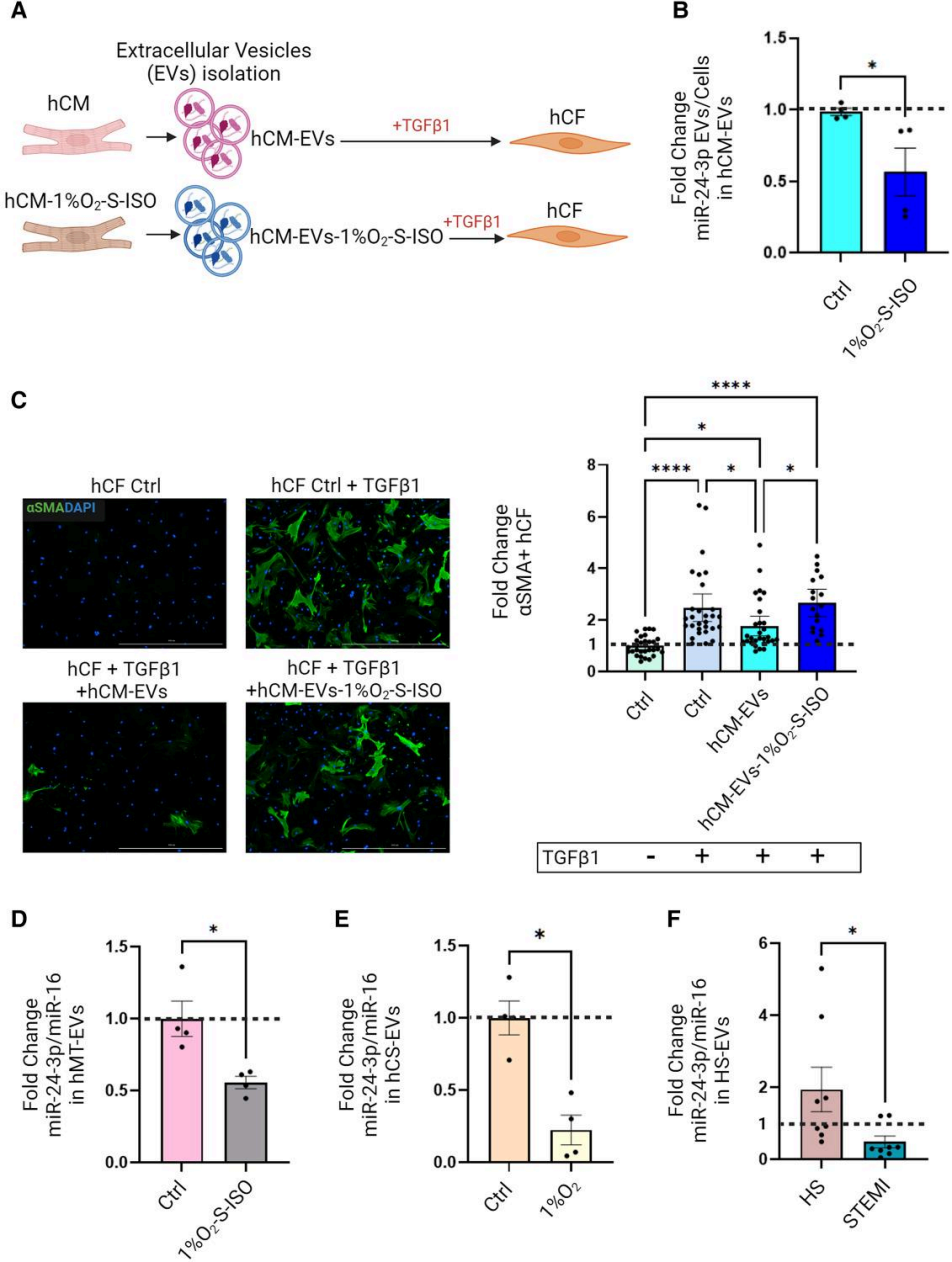
(*A*) Study design of comparisons of EVs secreted by hCM under normoxic (hCM-EVs) or ischaemia-like conditions [i.e. hypoxia (1% O_2_)/serum starvation (S)/500 µM ISO; (hCM-EVs-1%O_2_-S-ISO)]. (*B*) Real-time PCR analysis of miR-24-3p concentrations in hCM-EVs and hCM-EVs-1%O_2_-S-ISO. (*C*) Left: Representative immunocytochemical images of hCF incubated with either hCM-EVs or hCM-EVs-1%O_2_-S-ISO (αSMA, green, DAPI, blue; scale bar: 1000 µm). Right: Quantitative analysis of αSMA^+^ cell numbers of hCF treated with TGFβ1 and incubated with either hCM-EVs or hCM-EVs-1%O_2_-S-ISO. (*D*) ddPCR analysis of miR-24-3p concentrations in EVs secreted by human cardiac microtissues under either normoxic (hMT) or ischaemia-like conditions (hMT-1%O_2_-S-ISO; data normalized for miR-16 expression). (*E*) ddPCR analysis of miR-24-3p expression in EVs secreted by *ex vivo* cultured hCS under either normoxic (hCS) or hypoxic conditions (hCS-1%O_2_; data normalized for miR-16 expression). (*F*) Real-time PCR analysis of miR-24-3p expression in circulating EVs from healthy subjects (HS-EVs) and patients with AMI (STEMI-EVs; data normalized for miR-16 expression).

Having shown that hCM secrete miR-24-3p as EVs cargo, we assessed the impact of ischaemia-simulating conditions on this process (*Figure 7A*). hCM cultured under such conditions (see above) exhibited increased markers of cellular stress, but no significant changes in cell counts (see Supplementary material online, *Figure S7E and F*). They secreted higher numbers of EVs (hCM-EVs-1%O_2_-S-ISO) that were larger than those released from normoxic hCM (see Supplementary material online, *Figure S7G and H*). EVs from hCM cultured under ischaemia-simulating conditions showed lower miR-24-3p contents than same numbers of EVs from normoxic hCM (*Figure 7B*). The former failed to prevent TGFβ1-mediated hCF activation (*Figure 7C*). Moreover, EVs released from cardiac hMT cultured under ischaemia-simulating conditions exhibited lower miR-24-3p levels than those released from normoxic hMT (*Figure 7D*). Similar findings were observed for EVs secreted by *ex vivo* cultured human cardiac slices (hCS) under hypoxic conditions (*Figure 7E*). Finally, circulating EVs from patients with ST-elevation MI (STEMI), obtained within 3 h of pain onset [before percutaneous coronary intervention (PCI)], exhibited lower miR-24-3p contents than those from healthy subjects (*n* = 8/group; *Figure 7F*). Whether this observation reflects differences in miR-24-3p contents of cardiac-derived EVs between the two groups could not be assessed, however.

### 3.8 Pharmacological stimulation of miR-24-3p expression attenuates interstitial fibrosis in cardiac microtissues

**Figure 8:**
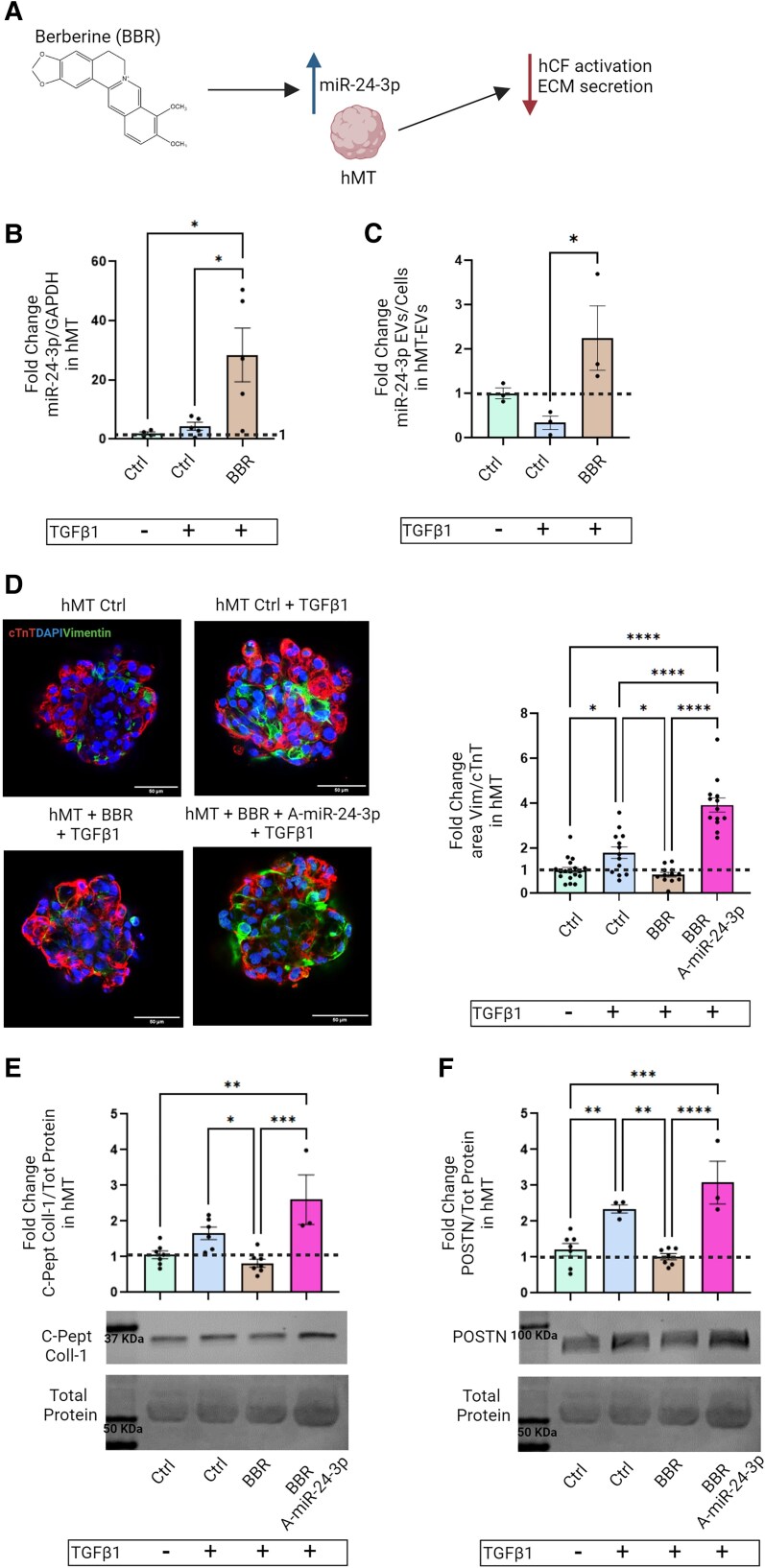
(*A*) Study design of BBR treatment of hMT. (*B*) Real-time PCR analysis of miR-24-3p expression in TGFβ1-treated hMT, with or without BBR treatment (data normalized for GAPDH expression). (*C*) ddPCR analysis of miR-24-3p expression in TGFβ1-treated hMT-EVs, with or without BBR treatment (data normalized for miR-16 expression). (*D*) Left: Representative immunofluorescence images of microtissues treated with TGFβ1, BBR, and A-miR-24-3p transfection (cTnT, red; Vimentin, green; DAPI, blue; scale bar: 50 µm). Right: Quantitative analysis of Vimentin^+^/cTnT^+^ area ratio in TGFβ1-treated hMT, with or without BBR treatment and A-miR-24-3p transfection. (*E*) Western analysis of C-peptide collagen-1 expression in conditioned media of TGFβ1-treated hMT, with or without BBR treatment and A-miR-24-3p transfection. (*F*) Western analysis of POSTN expression (data in *Figure 8B, D and F* are fold-changes for the indicated experimental conditions vs. naïve hMT). Total protein signal was used as a loading control.

Lastly, we tested the impact of pharmacological enhancement of miR-24-3p expression on CF activation in cardiac hMT. Previous reports showed that berberine (BBR), a natural phyto-compound, increased miR-24-3p expression in lymphoblastic leukaemia cells.^33^ Interestingly, BBR also reduced scar size in animal models of MI; however, its impact on miR-24-3p expression was not addressed by these studies.^34–36^ Here, BBR increased miR-24-3p levels both in TGFβ1-treated cardiac hMT (*Figure 8A and B*) and in secreted EVs (*Figure 8C*). These effects were associated with decreases in interstitial fibrosis, as evidenced by Vimentin^+^/cTnT^+^ area ratios (*Figure 8D*), and in c-peptide collagen-1 and POSTN expression (*Figure 8E, F*). Anti-miR-24-3p nullified BBR’s anti-fibrogenic effects (*Figure 8D–F*). Moreover, BBR suppressed TGFβ1-induced FURIN, CCND1, and SMAD4 up-regulation in hMT (see Supplementary material online, *Figure S9A–C*), supporting a miR-24-3p-dependent mechanism of action.

## 4. Discussion

The development of cardiac fibrosis in response to injury leads to myocardial stiffness and eventually heart failure. An improved understanding of the molecular mechanisms that regulate this process will be key to developing novel therapeutic approaches. miR-24-3p has been implicated in the regulation of cardiac fibrosis in animal models.^16–19^ Species-specific biological activities of miRs prompted us to assess the role of miR-24-3p in human cells and tissue samples. Using bioinformatic analyses of human databases,^20^ we showed that miR-24-3p is expressed at highest levels in normal human hearts, consistent with possible roles in cardiac homeostasis. By computation analysis of data from a spatial multi-omic map of human MI,^21^ we also showed that *FURIN*, *CCND1*, and *SMAD4*—three target genes for miR-24-3p involved in TGFβ1-activated fibrogenic pathways^22^—were differentially expressed by hCF after MI, when compared with hCF from normal hearts. These observations suggest that a miR-24-3p-dependent mechanism may inhibit fibrosis in normal human hearts, but not in injured ones.

Because primary adult human CM have major limitations as an *in vitro* model, hiPS-CM (hCM, for short) were used in the present study. These cells exhibit molecular and electrophysiological characteristics of mature CM, as reported previously.^37,38^ hCM expressed miR-24-3p at high levels, whereas primary adult hCF did not. This observation points to hCM as the main cellular source of miR-24-3p in normal hearts. Unlike naïve hCF, hCF transfected with a miR-24-3p mimic lacked FURIN, CCND1, and SMAD4 up-regulation in response to TGFβ1 treatment, which was associated with increased secretion of structural ECM and matricellular proteins, and increased hCF conversion into αSMA^+^ myofibroblasts. These findings raised the hypothesis that miR-24-3p secreted by hCM modulates hCF activation. To further investigate hCM-hCF cross-talk, we used a 3D cardiac microtissue (hMT) model of self-aggregating hCM, hCF, and hEC.^23^ Transfection of hCM with anti-miR-24-3p prior to the generation of hMT increased TGFβ1-mediated CF activation and interstitial fibrosis in this model. miR-24-3p down-regulated FURIN, CCND1, and SMAD4 in hMT.

Secreted EVs carry and deliver miRs, along with other nucleic acids, proteins, and lipids in their cargoes, to recipient cells. We therefore asked whether donor hCM could deliver miR-24-3p as an EV cargo to recipient hCF. We then performed digital PCR to quantify the number of miRs copies per EVs and found lower-than-expected levels (∼5 copies per 10⁸ EVs for miR-24-3p and ∼2 copies per 10⁹ EVs for miR-16). These results are likely influenced by the technical challenges of accurately determining EVs numbers via NTA and measuring miRs copy numbers per EV through PCR.^39^ Given the limitations of both methods, absolute quantification of miRNAs in individual EVs is unreliable and prone to variability. Recognizing this limitation, we have chosen to express miR-24-3p quantification as a relative value. Here, we found that, under normal conditions, hCM-EVs were enriched with miR-24-3p and prevented TGFβ1-induced hCF activation. hCM-EVs uptake by hCF was visualized using labelled EVs. Pharmacological inhibitors of EV release or cellular uptake abrogated inhibitory effects of hCM on hCF activation. Transfection of hCM with anti-miR-24-3p significantly reduced miR-24-3p concentrations in the secreted EV cargo and abrogated the ability of hCM-EVs to block TGFβ1-induced hCF activation in hMT. Moreover, transfection of hCF with anti-miR-24-3p rendered them refractory to the effects of naïve hCM-EVs.

We then addressed the regulation of cardiac fibrosis under *in vitro* conditions that simulate ischaemia. Under such conditions, hCM secreted EVs with markedly decreased miR-24-3p cargo levels, when compared with normoxic hCM, which failed to prevent myofibroblast activation and ECM protein secretion. Similar results were obtained using both cardiac hMT and *ex vivo* cultured hCS. Of note, the latter model includes primary hCM, as opposed to hiPS-derived CM. In addition, an RNAScope analysis of *post-mortem* heart tissue samples revealed a significant decrease in miR-24-3p expression in hCM from patients who died from MI, when compared with those who died from other causes in the absence of overt heart disease. Moreover, circulating EVs from patients with acute MI exhibited decreased miR-24-3p cargo levels, when compared with healthy subjects. Collectively, these results suggest that ischaemia may be associated with decreased miR-24-3p levels in hCM and their secreted EVs, resulting in loss of anti-fibrogenic activity. These results point to a novel paracrine mechanism by which hCM-derived, miR-24-3p-rich EVs are taken up by recipient hCF and block their activation. This mechanism is impaired by ischaemia, with pro-fibrogenic effects. A similar role was reported previously for umbilical mesenchymal stem cells (MSC)-derived, miR-24-3p-rich EVs.^40^ While this report is in line with our findings, umbilical MSC are irrelevant to the physiology of the adult heart. It is also worth noting that while previous studies used synthetic miR-24-3p precursors to assess functional activities of this miR, they did not address the source of endogenous miR-24-3p. Here, we identified CM as the main source of this miR in the heart, illustrating a novel potential aspect of CM–CF cross-talk. On the other hand, it should be emphasized that miR-24-3p, like many other miRs, exerts pleiotropic biological activities, e.g. anti-apoptotic^41^ and pro-angiogenic effects^42^ likely playing contributory roles in the response to cardiac injury. While a number of miRs have been implicated in the regulation of cardiac fibrosis,^43^ a significant role for miR-24-3p is suggested by its abundance in normal hearts.

In a translational perspective, we also performed a proof-of-concept study to explore the therapeutic usefulness of pharmacological miR-24-3p enhancement for preventing cardiac fibrosis. The pharmacological agent used was BBR, a natural cytoprotective phyto-compound that has been safely used in clinical studies of other disease conditions.^44^ Although beneficial effects of BBR in animal models of MI were described previously,^34,36^ the reported mechanism of benefit involved cytoprotection and immune modulation, and miR-24-3p expression was not measured. In a different biological setting, BBR was shown to stimulate miR-24-3p expression in lymphoblastic leukaemia cells.^33^ Here, we show that BBR likewise increases miR-24-3p expression in TGFβ1-treated hMT and their secreted EVs while decreasing ECM protein secretion and interstitial fibrosis in this model. These effects were associated with down-regulation of FURIN, CCND1, and SMAD4 in hMT. They were blocked by anti-miR-24-3p. While these observations support a role for miR-24-3p in anti-fibrogenic activities of BBR, other molecular mechanisms may also we involved.

## 5. Conclusions

Our findings suggest that EVs-mediated miR-24-3p delivery from hCM to hCF negatively regulates their activation and the development of cardiac fibrosis. The underlying mechanism includes *FURIN*, *CCND1*, and *SMAD4* gene targeting by miR-24-3p. Hypoxia/ischaemia down-regulates miR-24-3p in hCM, as confirmed by *post-mortem* analysis of myocardium from patients with MI, affecting this anti-fibrogenic mechanism.

## Supplementary Material

cvae243_Supplementary_Data

## Data Availability

The data underlying this article will be shared on reasonable request to the corresponding author.
