# Supplementary material for: miR-24-3p secreted as extracellular vesicle cargo by cardiomyocytes inhibits fibrosis in human cardiac microtissues
Source: Cardiovasc Res. 2024 Nov 11;121(1):143–56. doi: 10.1093/cvr/cvae243 (PMC11998913; doi:10.1093/cvr/cvae243)
Supplement: cvae243_Supplementary_Data [file cvae243_supplementary_data.docx]

**miR-24-3p secreted as extracellular vesicle cargo by cardiomyocytes inhibits fibrosis in human cardiac microtissues**

Giorgia Senesi,^1,2^, Alessandra M. Lodrini,^3^ Shafeeq Mohammed,^4^ Simone Mosole,^5^ Jesper Hjortnaes,^6^ Rogier J. A. Veltrop,^7^ Bela Kubat,^8^ Davide Ceresa,^9^ Sara Bolis,^1^ Andrea Raimondi,^10^ Tiziano Torre,^11^ Paolo Malatesta,^9,12^ Marie-José Goumans,^3^ Francesco Paneni,^4^ Giovanni G. Camici,^13^ Lucio Barile,^1,2,14^ Carolina Balbi,^13,15, a,^ * Giuseppe Vassalli^1,2,13^*

*^1^ Istituto Cardiocentro Ticino,* *Laboratories for Translational Research, Ente Ospedaliero Cantonale, Bellinzona, Switzerland;*

*^2^ Faculty of Biomedical Sciences, Università della Svizzera italiana, Lugano, Switzerland;*

*^3^ Department of Cell and Chemical Biology, Leiden University Medical Center, Leiden, the Netherlands;*

*^4^ Center for Translational and Experimental Cardiology, University Hospital Zürich and University of*

*Zürich, Zurich, Switzerland;*

*^5^ Institute of Oncology Research (IOR), Oncology Institute of Southern Switzerland (IOSI), Switzerland;*

*^6^ Department of Thoracic Surgery, Leiden University Medical Center, Leiden, the Netherlands;*

*^7^ Department of Biochemistry, Cardiovascular Research Institute Maastricht, Maastricht University, The Netherlands;*

*^8^ Department of Pathology, Maastricht University Medical Center, The Netherlands;*

*^9^ Cellular Oncology Unit, IRCCS Ospedale Policlinico San Martino, Genova, Italy;*

*^10^ Institute of Biomedical Research, IRB, Bellinzona, Switzerland*

*^11^ Heart Surgery Unit, Cardiocentro Ticino Institute, EOC, Lugano, Switzerland*

*^12^ Experimental Biology Unit, Department of Experimental Medicine (DIMES), University of Genova, Genova, Italy;*

*^13^ Center for Molecular Cardiology, University of Zurich, Schlieren, Switzerland;*

*^14^ Euler Institute, Faculty of Biomedical Sciences, Università della Svizzera italiana, Lugano, Switzerland;*

*^15^ Department of Medicine, Baden Cantonal Hospital, Baden, Switzerland.*

Present address:

*^a^* Carolina Balbi: *Center for Molecular Cardiology, University of Zurich, Schlieren, Switzerland;*

*These authors contributed equally to this study

Corresponding authors: Carolina Balbi, Center for Molecular Cardiology, Zurich, Switzerland

(carolina.balbi@uzh.ch)

Giuseppe Vassalli, Istituto Cardiocentro Ticino-EOC, Bellinzona,

Switzerland (giuseppe.vassalli@eoc.ch)

**Material and Methods**

1. ***Bioinformatic analysis***

**1.1 miRs analysis**

Analysis of miRs levels in human heart was performed using published data of Small RNA sequencing on healthy human heart samples ^1^.

**1.2 miR-24-3p target analysis**

Prediction of gene sequence targeted by miR-24-3p was performed using TargetScanHuman free web tool (<https://www.targetscan.org/vert_80/>).

**1.3 Heart gene expression analysis**

Gene expression of selected miR-24-3p target genes was performed by processing snRNA-seq data obtained from online available datasets ^2^ and also at cellxgene <https://cellxgene.cziscience.com/collections/8191c283-0816-424b-9b61-c3e1d6258a77>. All codes used for analysis are accessible at <https://github.com/saezlab/visium_heart> and <https://github.com/KramannLab/visium_heart>.

**1.4 STRING analysis**

STRING free web software (<https://string-db.org/>) was used to determine pathways involving SMAD4, FURIN and CCND1 proteins.

1. ***Samples***
   1. **Cell lines**

**All the protocols used in this study were approved by local Ethics Committee for Clinical Research (Comitato Etico Cantonale, Bellinzona, Switzerland; Rif. CE 2923).**

- - 1. **Human Cardiac Fibroblasts (hCF)**

Cardiac atrial appendage tissue samples were obtained from patients with no significant coronary artery disease, who underwent heart surgery for aortic regurgitation. hCF were obtained following enzymatic dissociation of atrial specimens. Cardiac-derived cells were cultured and maintained *in vitro* in EGM-2 complete medium, composed of EBM^TM^-2 Basal Medium and EGM^TM^-2 SingleQuots^TM^ Supplements (CC-3156, Lonza, CH). Culture medium was changed twice a week. For experiments, hCF were enzymatically detached using Trypsin/EDTA (25200056, Gibco, USA) at 37 °C, which was subsequently blocked with culture medium containing 20% FBS. Cells were then centrifuged at 1100 rpm for 5 min, re-suspended, and plated (1.5 × 10^4^ cells/cm^2^). Cells were used for experimentation 24hrs after plating.

- - 1. **Human Cardiomyocytes (hCM)**
       1. **iPS-cell reprogramming**

Induced Pluripotent Stem (iPS) cells were obtained by reprogramming human cardiac mesenchymal progenitor cells (CPC) as previously described by our group ^3,4^. Briefly, CPC were infected with Sendai virus carrying OCT3/4, SOX2, KLF4, and MYC (CytoTune™-iPS 2.0 Sendai Reprogramming Kit, A16517, Thermo Fisher Scientific, USA), as per manufacturer’s instructions. A week after transduction, the medium was changed to StemFlex (A3349401 - Thermo Fischer Scientific, USA). Individual colonies with embryonal stem cells (ESC)-like morphology typically appeared after 25–35 days and were transferred and expanded into Matrigel (hESC Qualified Matrix, 354277, Corning, USA) coated wells. Established human iPS cell lines were maintained in culture in StemFlex medium.

- - - 1. **Differentiation of iPS cells into iPS-derived cardiomyocytes (hCM)**

Directed differentiation of human iPS cells into human cardiomyocytes (hCM) was performed using StemMACS™ CardioDiff Kit XF (130-125-289 - Miltenyi Biotec, DE), as per manufacturer’s instructions. The medium was changed to a maintenance medium composed of RPMI 1640 (11875093, Gibco, USA) with B-27 plus insulin (17504044, Thermo Fisher Scientific, USA) at day 7. Metabolic selection of hCM was performed using a selection medium composed of RPMI 1640 without glucose (11879020, Gibco, USA), 0.5 mg/ml human recombinant albumin (44206-310, CSL Behring AG, CH), 0.2 mg/ml L-ascorbic acid 2-phosphate (05878, Fluka, USA) and 4 mM lactate (Sigma-Aldrich, USA) from day 10 to day 17. Subsequently, hCM were cultured in a maintenance medium for at least 30 days for further maturation. For experiments, hCM were enzymatically detached using Multi-tissue-dissociation kit (ET) (130-110-204, Miltenyi Biotec, DE) at 37 °C, which was subsequently blocked with culture medium containing 20% FBS (16000044, Gibco, USA). Cells were then centrifuged at 1100 rpm for 5’, re-suspended, and plated at 6 × 10^5^ cells/cm^2^. Cells were used for experimentation 24hrs after plating.

- - 1. **Human Aortic Endothelial Cells (hAEC)**

human Aortic Endothelial Cells hAEC (CC-2535, Lonza, CH) were cultured and maintained *in vitro* in EGM-2 complete medium supplemented with 10% FBS. For experiments, hAEC were enzymatically detached using TrypLE™ Express Enzyme 1X (12605010, Gibco, USA) at 37 °C, and subsequently blocked with culture medium containing 20% FBS. Cells were then centrifuged at 1100 rpm for 5’, re-suspended, and plated (1.3 × 10^4^ cells/cm^2^). Cells were used for experimentation 24hrs after plating.

- - 1. **Human 3D microtissues (hMT)**

Human 3D microtissue (hMT), composed of hCM, hCF, and hEC in the proportion of 1.0:0.2:0.2 (3500:750:750 cells) were generated using low-binding BIOFLOAT™ 96-well plates (faCellitate, DE). After a centrifugation at 1100 rpm for 5’, hMT were cultured and maintained *in vitro* in EGM-2 complete medium. Two days after plating, hMT were spontaneously beating and ready for experiments.

- 1. **Tissue samples**
     1. **Human Cardiac Slices (hCS)**

**Culture and treatment of human cardiac slices (hCS) were performed in collaboration with Prof. Marie-Josè Goumans (Leiden University Medical Center). This study was conducted in accordance with the Ethical Principles of the Declaration of Helsinki 2013 and according to the Dutch regulation for responsible use of human tissues for medical research purposes. The institutional Medical Ethics Committee ruled that the Medical Research Involving Human Subject Act (WMO) does not apply to the use of surgical waste material (reference number B12.017).**

Myocardial samples were obtained from surgical waste material of patients who underwent valve replacement surgery or Morrow myectomy. Myocardial slices were obtained and cultured as previously described ^5,6^. Briefly, left ventricular samples were cut into 1 cm^3^ blocks. Each heart tissue cube was embedded in 4% low-gelling temperature agarose solution and glued using Histoacryl surgical glue (Braun, DE cat. No. 1050052) on top of the specimen holder of a Vibrating Microtome (VT1200S, Leica, DE). The cutting chamber was filled with cold (4°C) modified Tyrode’s solution (NaCl 140 mM: KCl 6 mM; CaCl2 1.8 mM; MgCl2 1 mM, Hepes 10 mM; Glucose 1 mM; 2,3-butanedione monoxime (BDM) 30mM; pH 7.4). Slices were then cut with 300μm slice thickness, 0.03 mm/s advance speed, and 2mm horizontal vibration amplitude. Following slicing, each slice was washed with Tyrode’s solution at RT (NaCl 140 mM: KCl 6 mM; CaCl2 1.8 mM; MgCl2 1 mM, Hepes 10 mM; Glucose 1 mM; 3% Penicillin-Streptomycin; pH 7.4) and placed on semi-porous transwell inserts (PICM0RG0, Millipore, USA) in 6-well plate format with 1 mL culture medium (Medium-199; 0.001% ITS liquid media supplement; 1% Penicillin-Streptomycin) to obtain a liquid-air interface. Slices where then incubated at 37°C with 5% CO^2^. Culture medium was replaced every 48 hours.

- - 1. **Human cardiac tissue**

**Human heart samples were collected at the Department of Pathology, Maastricht University Medical Center, The Netherlands. Sample collection was approved by local ethical committee (METC) number 21-017.**

Heart samples were obtained from 5 different patients who died after acute myocardial infarction, and 4 control tissues obtained from subjects who died for various reasons not related to heart disease including pneumonia, pancreatitis, and peritonitis. All samples were obtained within 48 hours (average: 29 hours) after death.

- 1. **Plasma samples**

**Protocols used in this study were approved by local Ethics Committee for Clinical Research (Comitato Etico Cantonale, Bellinzona, Switzerland; Rif. CE 2224).**

**All participants gave informed written consent to the study in accordance with the declaration of Helsink**i **2013**.

We analysed peripheral venous blood samples collected from individuals recruited at the Istituto Cardiocentro Ticino, Lugano (Switzerland). Peripheral blood samples were collected from patients presenting with a diagnosis of STEMI, according to the European Society of Cardiology (ESC) guidelines ^7^, at the emergency department before primary percutaneous coronary intervention (PCI). Exclusion criteria included: (a) Chest pain onset ≥6 hours; (b) Age > 85; (c) Cardiac arrest or cardiogenic shock with indication to invasive device assistance, as described previously. ^8^.

1. ***Extracellular vesicles isolation***

**EVs isolation was performed in accordance to MISEV guidelines** ^9^**.**

- 1. **Collection**
     1. **Conditioned medium**

Cell conditioned medium was collected and centrifuged at 3.000 *g* for 15’, and cell debris were discarded. Supernatant was then centrifugated at 10.000 *g* for 15 min to remove larger vesicles, EV aggregates and apoptotic bodies (see supplementary Figure 5A).

- - 1. **Serum**

Blood was collected in 7 mL heparin‐ and EDTA‐free polypropylene tubes. The first blood tube was discarded. Blood was centrifuged at 1600g for 15 min at 4°C, and supernatant was centrifuged at 3.000 *g* for 20’, 10.000 *g* for 15’, and 20.000 *g* for 30 min to remove intact cells, cellular debris and larger EVs.

- 1. **Isolation**

EVs from pre-cleaned conditioned media or serum were isolated using Size Exclusion Chromatography (SEC). SEC was performed using commercially available columns (q70nm column-Izon, NZ), according to manufacturer’s instructions. Pre-cleaned sample was added to the top of the column and then eluted using PBS 1X. Fractions 7-8-9-10 enriched in EVs were collected and subjected to a second isolation step by ultracentrifugation (UC), performed at 100.000 *g* for 70 min at 10°C. The obtained pellet was resuspended in 100 µL PBS 1x, and then analysed by Nanoparticle Tracking Analysis (NTA), Transmission Electron Microscope (TEM), and Fluorescence-activated cell sorting (FACS).

- 1. **Analysis**

**3.3.1 Nanoparticle Tracking Analysis**

Nanoparticle Tracking analysis (NTA) measurements were carried out with Zetaview (Particle Metrix). Shortly, 1 μL of isolated EVs was diluted in PBS 1x to a final volume of 500 μL. The manufacturer’s default software settings for EVs or nanospheres were selected accordingly. For each measurement, 11 cell positions with a cell temperature of 25 °C were acquired. After capture, videos are analysed by the in-build ZetaView Software 8.02.31. Hardware: embedded laser: 40mW at 520nm; camera: CMOS. The number of completed tracks in NTA measurements was always greater than the proposed minimum of 1.000 in order to minimize data skewing based on single large particles. Particle numbers and size (diameter) were expressed in number/mL and nm, respectively.

**3.3.2 Transmission Electron Microscope (TEM)**

Morphological evaluations of isolated hCM-derived EVs were performed using Transmission Electron Microscopy (TEM) negative staining. EVs resuspended in 100μL of PBS 1x were diluted 1:100 and absorbed on a glow-discharged carbon-coated formvar copper grid and negatively stained with 2% uranyl acetate. EVs pictures were examined by a Talos L120C (FEI, Thermo Fisher Scientific) operating at 120 kV. Images were acquired with a Ceta CCD camera (FEI, Thermo Fisher Scientific).

**3.3.3 Fluorescence-activated cell sorting (FACS)**

CD9, CD63 and CD81 (JSR Life Science) expression was investigated by flow cytometry. as previously described ^10^. Briefly, 1*10^8^ hCM-derived EVs resuspended in 100μL of PBS 1x were incubated overnight at 10 °C and 400 rpm with 1 μL of CD9, CD63, and CD81 (JSR Life Sciences Ex-C9-SP; Ex-C63-SP; Ex-C81-SP; ratio 1:1:1) mixed beads, corresponding to 1.2*10^5^ beads in total (for each test). After 24h, 1μL of 10μg/mL of FITC-conjugated CD9 (Biolegend 312104) or 10 μg/mL of PE-conjugated CD63 (Biolegend 353004) or 5 μg/mL of PE-conjugated CD81 (Biolegend 349505) or 10μg/mL of PE-conjugated CD105 (Biolegend 323205) or 5μg/mL of APC-conjugated CD172a (Milteny 130-133-369) was added to 100 μL of CM derived-EVs and bead-containing samples, which were then acquired (20.000 events) with CytoFLEX (Beckman Coulter) and analysed using Kaluza software (Beckman Coulter).

1. ***Cell treatments***
   1. **TGFβ1 treatment**

To induce activation of hCF into myofibroblasts, hCF and hMT were treated with TGFβ1 (CYT-716, Prospect, UK) at 10 ng/mL concentrations in culture media. Treatment read-out was performed at 72 hrs for FURIN, CCND1 and SMAD4 analysis, and at 7 days (with medium change at day 4, with no TGFβ1 replacement) for analysis of structural proteins (αSMA) and proteins involved in extracellular matrix (ECM) production (periostin and collagen-1). TGFβ1 concentration in hCF-conditioned media was determined using Human TGFβ1 ELISA Kit (Abcam, ab100647, USA) after 72 hrs of treatment, as per manufacturer’s instructions.

- 1. **Berberine treatment**

Berberine chloride (BBR, C20H19NO5, a 5,6-dihydro-dibenzo[a,g]quinolizinium derivative; Y0001149, Sigma-Aldrich, USA) was used to stimulate miR24-3p up-regulation. hMT and hCM were treated with BBR (100µM) diluted in methanol (for HPLC, gradient grade, ≥99,9%, bioavailability 0.68%, CAS 67-56-1; 34885-1L-R, Sigma-Aldrich, USA). The dilution was resuspended in complete media, with TGFβ1, if necessary to induce activation, for 7 days (medium was changed at day 4, with no addition of TGFβ1 or BBR).

- 1. **miR mimic / anti-miR transfection**

To investigate the role of miR-24-3p, hCF or hMT were transfected with either miR-24-3p (*mir*Vana® miRNA mimic 4464066; ThermoFisher Scientific, USA) or anti-miR-24-3p (*mir*Vana® miRNA inhibitor 4464084; ThermoFisher Scientific), at a final concentration of 50 nM in F12 medium (11765054, Gibco, USA) supplemented with Lipofectamine RNAiMAX Reagent (13778075, Invitrogen, USA), as per manufacturer’s instructions. After 1 hr, cells were washed and medium replaced with maintenance medium. For hCM transfection, cells were transfected with anti-miR-24-3p. Transfections were performed using jetPRIME^®^ (101000046, Polyplus, FR), as per manufacturer’s instructions. After 1 hr, hCM were pelleted down with hCF and hAEC and co-cultured under hMT conditions. TGFβ was added, if necessary, to stimulate hCF activation. MiR scramble transfection with mirVana™ miRNA Negative Control (4464058, Ambion, USA) was used as a control, applying the conditions described above.

- 1. **Ischaemia-simulatiung conditions**

To mimic ischaemia *in vitro*, hCM and hMT were cultured under hypoxic conditions (1% O_2_ and 5% CO_2_) in the absence of serum, and treated with 500 µM isoprenaline hydrocholoride (I5627-1G, Sigma-Aldrich, USA) according to previously described protocols ^11,12^ , during 24 hrs for hCM and 7 days for hMT. hCS were cultured in a hypoxic chamber (1% O2 and 5% CO2) for 7 days, based on a previously validated protocol.

- 1. **hCM pre-conditioning for EVs enrichment**

hCM were cultured for 24 hrs under starvation conditions (RPMI) to avoid lipoprotein contamination. To re-establish physiological conditions while avoiding any intracellular modification, starvation media was replaced with RPMI + B27 (without serum), and conditioned media was collected after 24 hrs. EVs were isolated from conditioned media as described above (section 3: EV isolation).

- 1. **hCM-EV treatment**

hCF were treated with a concentration of 1*10^6^ EVs/cm^2^ hCM-EVs.

- 1. **GW4869 treatment**

To block EV biogenesis, hCM were treated with 20 µM of GW4869 (Sigma-Aldrich, USA). GW4869 was previously described to block EVs biogenesis ^13^ and frequently used for this purpose using *in vitro* or *in vivo* models ^14^.

- 1. **Blocking EV uptake**

Two compounds were used to block EV uptake in hCF: Dynasore and Heparine. Dynasore (Sigma-Aldrich, USA) was used to block EV endocytosis from cells’ plasma membranes ^15^. Briefly, we pre-treated hCF with 80 µM of Dynasore diluted in culture medium for 30 min at 37°C. Next, cells were washed, fresh medium was added, and cells were incubated with EVs. Heparin (Grosse Apoteke, Switzerland) was used to bind EVs in the media, thereby blocking their uptake by recipient cells, as previously described ^16,17^. hCF was pre-treated with 20 µg/mL of heparin diluted in culture medium for 30 min at 37°C. Next, cells were washed, fresh medium was added, and cells were incubated EVs.

- 1. **Fluorescent labelling of EVs**

hCM conditioned media (500 µL) was labelled with 5 µL of lipophilic commercial dye DiR (5 mg/mL; Thermo Fisher Scientific, USA) and incubated at 37°C for 5 min under gentle shaking. EV were then isolated by SEC followed by UC, as described above (section 3. EV isolation). hCF were then treated with DiR labeled EVs, and live images of cells were acquired 24 hrs later.

1. ***Western blotting***
   1. **Cell analyses**

Total proteins were extracted by lysing cells with ice-cold RIPA buffer 1X (89901, ThermoFisher Scientific, USA) supplemented with SIGMAFAST™ Protease Inhibitors (S8820, Sigma-Aldrich, USA) and then mixed at 1000 rpm at 10°C for 30 min to disrupt cells membrane. For hMT only, 1 min. sonication was performed. After that, proteins from all cellular compartments were centrifuged at 9.600 rpm for 15 min to discard protein membranes. Quantipro™ BCA Assay Kit (QPBCA, Sigma-Aldrich, USA) was used for protein concentration detection. Protein were denaturated and reduced at 95°C for 5 min with Laemmli SDS sample buffer 6× containing: 0.375 M Tris-HCl pH 6.8, 12% SDS, 60% glycerol, 0.6 M DTT, 20% (v/v) beta-mercaptoethanol, 70.2% (w/v) bromophenol blue (J60015, VWR International LCC, USA). Proteins were separated on 4-20% Mini-PROTEAN® TGX™ Precast Gel (4561094**,** Bio-Rad, USA) and transferred onto a Trans-Blot Turbo PVDF membrane with a semi-dry transfer system (1704156, Bio-Rad, USA). The membranes were blocked for 40 min with Intercept (TBS) Sure Block (SB232010-250G, LubioScience, CH) and incubated with the primary Abs at 4°C overnight (anti-GAPDH, 1:10.000, ab181602; anti-Furin, 1:200, Bio-Techne Sales Corp., AF1503; anti-CyclinD1, 1:10.000, ab134175; anti-SMAD4, 1:500, ab40759; anti-CD105, 1:1000, ab231774; anti-Vimentin, 1:2500, ab92547).

Membranes were then rinsed and incubated with appropriated fluorophore-conjugated secondary antibodies (Li-COR, USA) at RT for 2hrs in a concentration of 1:15.000. Membranes were then rinsed, acquired and analysed using Odyssey CLx Detection System (LI-COR Biosciences).

- 1. **Conditioned medium analyses**

Conditioned media obtained from treated cells were centrifuged at 3000 *g* for 15 min to discard cells debris. For each experiment, same volumes of conditioned media were analysed. Proteins in the media were denaturated and reduced using the conditions described above for cell analyses, and Western blotting was performed described above. After transfer, the membrane was stained with Revert Total protein Stain (LI-COR Biosciences, 926-11011) to determine total protein distribution, used for protein normalisation. Membranes were then rinsed with revert total protein wash buffer (6,7% glacial acetic acid and 30% methanol diluted in ddH_2_O glacial acetic acid; Carlo Erba, 401392) and acquired with Odyssey CLx Detection System (LI-COR Biosciences). After acquisition, membranes were blocked and primary Abs added overnight at 4 °C. Primary Abs used for conditioned media were as follows: anti-Periostin, (1:300, Santa Cruz Biotechnology, sc 398631); anti-Collagen I C-Pro Peptide (1:1.000, Abcam, ab255809).

- 1. **EVs analysis**

To evaluate tetraspanin (CD63 and CD81) presence on EVs, vesicles were loaded on 4-20% Mini-PROTEAN® TGX™ Precast Gel (4561094, Bio-Rad, USA) using non-reducing and non-denaturating conditions. For all other markers, EVs were treated as cell lysates and Western blot analysis performed as described above (section 5.1). Primary Abs used for analysis were as follows: anti-CD63 (1:1.000, Invitrogen, 10628D); anti-CD81 (1:1.000, BD, 555675); anti-TSG101 (1:1.000, Abcam, ab125011); anti-Syntenin 1 (1:1.000, Abcam, ab19903); and anti-GRP94 (1:1.000, Abcam, ab238126).

1. ***Dot Blots***

Isolated EVs were loaded into Immun-Blot PVDF membrane (162-0238, Bio-Rad, USA), previously activated with methanol. Once the membrane absorbed the sample, 15 min blocking (SureBlock LubioScience, SB232010-500G, CH) was performed. Primary Ab (anti-CD63, 1:1.000; Invitrogen, 10628D) and secondary Ab were used as described above (Section 5.1).

1. ***RNA extraction***

To analyse miR-24-3p expression in cells, hMT were pelleted at 1.200 rpm for 5'. Pellets were resuspended in 1 mL of TRI reagent (TR118100, Genbiotech, BRA). After vortexing, 200 µL of chloroform (151831, Sigma-Aldrich, USA) was added and samples shekered at 1.500 rpm for 10 min at 4 °C. To remove lipids and proteins separated by chloroform, samples were centrifuged at 12.000 rpm for 15 min at 4 °C. The aqueous phase containing RNA was then transferred into 500 µL of 2-propanol (34863, Sigma-Aldrich, USA) to ensure precipitation, with the addition of 1 µL of glycoblue (nucleic acid co-precipitant) for 30 min at -80 °C. Samples were then centrifugated at 1.200 rpm for 30 min at 4 °C. The pelleted RNA was washed two times in 75% ethanol and subjected to 7.500 rpm centrifugation for 5 min at 4 °C to allow the RNA to hydrate. Pellet was air-dried, re-suspended in DEPC water, and RNA was quantified with NanoDrop™ 2000c (Thermo Fisher Scientific,USA).

1. ***Real-Time PCR***
   1. **miR reverse transcription and qPCR**

Reverse transcription for miRs was performed using TaqMan™ MicroRNA Reverse Transcription Kit, as per manifacturer’s instructions. This kit performs a specific retro-transcription for the miR of interest. Here, miR16 and miR-24-3p were measures (Taqman™ MicroRNA Assay hsa-miR-16, Thermo-Fisher, 4440887 000391, and Taqman™ MicroRNA Assay hsa-miR-24, Thermo-Fisher, 4440887 000402, respectively). Real-time analysis was performed on CFX Connect™ Real-Time PCR Detection System (Bio-Rad, USA) using TaqMan (Thermo Fisher Scientific, USA) and specific primers (Taqman™ MicroRNA Assay hsa-miR-16, Thermo-Fisher, 4440887 000391; Taqman™ MicroRNA Assay hsa-miR-24, Thermo-Fisher, 4440887 000402).

- 1. **mRNA reverse transcription and qPCR**

Total RNA for GAPDH evaluation was reverse-transcribed using GoScript™ Reverse Transcription System (Promega Madison, Dübendorf, Switzerland) as per manufacturer’s instructions. To perform real-time PCR, the following mix was used: 2 μL DEPC water, 5 μL SsoAdvanced Universal SYBR Green Supermix 2x (BioRad), 2 μL of cDNA diluted 1:5 in DEPC water, 0.5 μL GAPDH primer forward 10mM and 0.5 μL GAPDH primer reverse 10mM (provided by Microsyinth AG, CH). Amplification and detection of specific products were performed in triplicates using a CFX Connect™ Real-Time PCR Detection System (Bio-Rad, USA). Primers were as follows: Human *GAPDH*-forward: TGCACCACCAACTGCTTAGC; Human *GAPDH*-reverse: GGCATGGACTGTGGTCATGAG. Results are shown as 2^-ΔΔCt^ values.

1. ***Digital-PCR (ddPCR)***

ddPCR was performed to assess miR-24-3p copy numbers in hCM-EV cargoes, and generally to quantitatively assess miR-24-3p levels using small amounts of molecular material (e.g., hMT conditioned media). ddPCR was performed using tDroplet Digital™ PCR kit (ddPCR™, Bio-Rad, USA), as per manufacturer's instructions. After specifically retro-transcribing miR-24-3p or miR16, ddPCR analysis was performed on 2 µL of a 1:20 dilution for hCS; 5 µL of hCM-EVs; 7 µL of hMT-EVs. The specific probe (1 µL) used was derived from the kit TaqMan™ MicroRNA Reverse Transcription Kit (Taqman™ MicroRNA Assay hsa-miR-16, Thermo-Fisher, 4440887 000391; Taqman™ MicroRNA Assay hsa-miR-24, Thermo-Fisher, 4440887 000402). Amplification and detection of specific products were performed using a Droplet Digital™ PCR (ddPCR™, Bio-Rad, USA) system. Results were calculated by defining the miR concentration (copies) in total RNA or in each µl of EVs.

1. ***Immunofluorescence***

After treatment, cells or hMT were fixed for 5 min at RT using a PFA-4%. Fixed cells were then washed with PBS 1x for 3 times. Cells were then permeabilised with 0.3% Triton X (Triton X detergent, Sigma-Aldrich) and blocked in 2% bovine serum albumin (BSA; Merck, USA) in PBS 1x for 40 min at RT, followed by PBS 1x washing for 5’.

- 1. **Immunostaining of cells**

hCM and hCF were incubated with PBS 1x containing 0.3% Triton X, 1% BSA, and the primary Ab overnight at 4°C. To assess myofibroblast activation, hCF were stained using Ab against α-smooth muscle actin (α-SMA, 1:250, 19245, Cell Signaling Technology, USA). For hCM characterisation, cells were stained for cardiac troponin T (cTnT, 1:300, 564766, BD Biosciences, USA). Cell damage under hypoxia conditions was assessed by staining cells for γH2AX (1:500, 9718, Cell Signaling Technology, USA). After incubation with the primary Av, cells were washed 3 times in PBS 1x and incubated for 2hrs at RT with appropriate fluorophore-conjugated secondary Abs (Alexa Fluor-488; Alexa Fluor-594 and Alexa Fluor-633 1:400, Thermo Fisher Scientific, USA) and DAPI (1:1000 10236276001, Roche, CH) in PBS 1x, 0.3% Triton X, 1% BSA.

- 1. **Immunostaining of hMT**

hMT were incubated for 2 days at RT with PBS1 x containing 0.3% Triton X, 1% BSA and the respective primary Abs, under gentle shaking. Primary Abs were as follows: Cardiac Troponin T for hCM (see section 10.2.1); Vimentin for hCF (1 :500, Abcam, ab92547); CD31 for hAEC (1:1.000, Abcam, ab9498). To asses hCM damage hMT were stained for γH2AX (see section 10.2.1). hMT were washed with PBS 1x and secondary Abs (see section 10.2.1) were incubated for 1 day at RT. hMT were then washed with PBS 1s for 3 times and included for imaging into chamber slides in 1% low melt agarose (H26417-14, Thermo Fisher Scientific, USA).

1. ***Immunofluorescence for hCS and human heart tissue***

hCS for immunofluorescence analysis were fixed in 4% PFA for 4 hrs at RT under gentle shaking, and then embedded in paraffin for sectioning. Heart tissue samples were fixed in 10% buffered formalin for 5 days. Next, the infarcted left ventricular zone (or the equivalent in control hearts) was embedded in paraffin. hCS and heart tissue were cut in 4µm slice and subjected to immunofluorescence analysis and RNAScope analysis.

- 1. **TUNEL-assay and lipofuscin detection**

Cell death was analysed using TUNEL-assay (In Situ Cell Death Detection Kit, Fluorescein, 11684795910, Roche, Switzerland), as per manufacturers’ instructions. hCS were then counter-stained with DAPI (1:1.000, 10236276001, Roche). For stress analysis, hCS were analysed to detect endogenous lipofuscin-like autofluorescence. hCS were counterstained with wheat germ agglutinin-Alexa Fluor™ 633 (1:100, WGA, W11261, Thermo Fisher Scientific, USA).

- 1. **RNA Scope**

RNAscope for miR-24-3p and TNNT was achieved according to the manufacturer’s protocol (RNAscope Plus smRNA-RNA HD) followed by an incubation for WGA (Thermo Fisher 1:100). First, slides were baked for 30 min at 60 °C and post-fixed in 10% NBF overnight at RT. For the pre-treatment steps, sections were covered for 10 min at RT with RNAscope Hydrogen Peroxide followed by 15 min in mid boiling RNAscope Target Retrieval Reagents (ACDBio 322000), and then incubated for 30 min at 40 °C with RNAscope Protease III (RNAscope H2O2 and protease reagents ACDBio Cat 322381). A mix of the probes for mir24-S1 (RTU ACDBio cat 893321-S1) and TNNT-C2 (1:50 ACDBio 518991-C2) was prepared according to the manufacturer’s instructions and used to incubate the samples for 2 hrs at 40 °C. The following steps of Amplification and Development of the signal were also performed following exactly the RNAscope Plus smRNA-RNA HD Reagent Kit Assay protocol (ACDBio Cat 322785). TSA Vivid 520 for TNNT (1:1.000 ACDBio 323271) and TSA Vivid 570 for miR-24-3p (1:1.000 ACDBio 323272) were used as fluorophores. Prior to DAPI staining, sections were subjected to the WGA protocol, avoiding direct light exposure. After DAPI staining (RNAscope Multiplex FL v2 DAPI ACDBio RTU Cat 323110), slides were cover-slipped with ProLong Gold anti-fade reagent with DAPI (Invitrogen P36931), and air-dried overnight at RT.

1. ***Imaging analysis***

**12.1** **Automated microscope**

Images of hCF were acquired using the BioTek Lionheart FX automated microscope (Biotek, USA) at 4× magnification and analysed with Gen5 software (BioTek, USA). A first mask on DAPI cells was created for cell counts; then mean fluorescent intensity (MFI) of the αSMA signal was defined to automatically count αSMA^+^ cells. For DiR analysis, acquisition was performed with heated chamber (37°C) and analysis was performed as follows: first mask on DAPI cells was created for cell identification; then MFI of the DiR signal was analysed.

**12.2** **Confocal microscope**

Samples were imaged on the Stellaris 5 Confocal Microscope (Leica). For γH2AX, detection images were acquired using an oil 63x magnification, while vimentin images were taken at 20x magnification. Images were acquired with as Z-stacks. Analyses was processed using ImageJ (<https://imagej.download.it/>) software. Same threshold was used for all samples.

1. ***Statistical analysis***

Data were generated using Prism Version 9 GraphPad Software. They were expressed as mean ± standard error of the mean (SEM) of independent experiments. The differences between groups were tested with unpaired t-test or one-way ANOVA analysis, as appropriate. Post-hoc comparison between individual means was performed with Tukey test. For data obtained using the hCS model, differences between groups were analysed with paired t-test (paired comparison of slices derived from the same patient). A *p*-value <0.05 was considered statistically significant (*, *p*<0.05; **, *p*<0.01; ***, *p*<0.001; ****, *p* <0.0001). Outlier data was defined using Outlier Calculator (<https://www.graphpad.com/quickcalcs/Grubbs1.cfm> ) and not considered for statistical analysis (p<0.05).

**References:**

1. Leptidis S, El Azzouzi H, Lok SI, Weger R de, Olieslagers S, Kisters N, Silva GJ, Heymans S, Cuppen E, Berezikov E, De Windt LJ, Costa Martins P da. A deep sequencing approach to uncover the miRNOME in the human heart. *PLoS One* 2013;**8**:e57800.

2. Kuppe C, Ramirez Flores RO, Li Z, Hayat S, Levinson RT, Liao X, Hannani MT, Tanevski J, Wünnemann F, Nagai JS, Halder M, Schumacher D, Menzel S, Schäfer G, Hoeft K, Cheng M, Ziegler S, Zhang X, Peisker F, Kaesler N, Saritas T, Xu Y, Kassner A, Gummert J, Morshuis M, Amrute J, Veltrop RJA, Boor P, Klingel K, Van Laake LW, Vink A, Hoogenboezem RM, Bindels EMJ, Schurgers L, Sattler S, Schapiro D, Schneider RK, Lavine K, Milting H, Costa IG, Saez-Rodriguez J, Kramann R. Spatial multi-omic map of human myocardial infarction. *Nature* 2022;**608**:766–777.

3. Pianezzi E, Altomare C, Bolis S, Balbi C, Torre T, Rinaldi A, Camici GG, Barile L, Vassalli G. Role of somatic cell sources in the maturation degree of human induced pluripotent stem cell-derived cardiomyocytes. *Biochim Biophys Acta Mol Cell Res* 2020;**1867**:118538.

4. Altomare C, Pianezzi E, Cervio E, Bolis S, Biemmi V, Benzoni P, Camici GG, Moccetti T, Barile L, Vassalli G. Human-induced pluripotent stem cell-derived cardiomyocytes from cardiac progenitor cells: effects of selective ion channel blockade. *Europace* 2016;**18**:iv67–iv76.

5. Watson SA, Scigliano M, Bardi I, Ascione R, Terracciano CM, Perbellini F. Preparation of viable adult ventricular myocardial slices from large and small mammals. *Nat Protoc* 2017;**12**:2623–2639.

6. Ou Q, Jacobson Z, Abouleisa RRE, Tang X-L, Hindi SM, Kumar A, Ivey KN, Giridharan G, El-Baz A, Brittian K, Rood B, Lin Y-H, Watson SA, Perbellini F, McKinsey TA, Hill BG, Jones SP, Terracciano CM, Bolli R, Mohamed TMA. Physiological Biomimetic Culture System for Pig and Human Heart Slices. *Circ Res* 2019;**125**:628–642.

7. Ibanez B, James S, Agewall S, Antunes MJ, Bucciarelli-Ducci C, Bueno H, Caforio ALP, Crea F, Goudevenos JA, Halvorsen S, Hindricks G, Kastrati A, Lenzen MJ, Prescott E, Roffi M, Valgimigli M, Varenhorst C, Vranckx P, Widimský P, ESC Scientific Document Group. 2017 ESC Guidelines for the management of acute myocardial infarction in patients presenting with ST-segment elevation: The Task Force for the management of acute myocardial infarction in patients presenting with ST-segment elevation of the European Society of Cardiology (ESC). *Eur Heart J* 2018;**39**:119–177.

8. Burrello J, Bolis S, Balbi C, Burrello A, Provasi E, Caporali E, Gauthier LG, Peirone A, D’Ascenzo F, Monticone S, Barile L, Vassalli G. An extracellular vesicle epitope profile is associated with acute myocardial infarction. *J Cell Mol Med* 2020;**24**:9945–9957.

9. Welsh JA, Goberdhan DCI, O’Driscoll L, Buzas EI, Blenkiron C, Bussolati B, Cai H, Di Vizio D, Driedonks TAP, Erdbrügger U, Falcon-Perez JM, Fu Q-L, Hill AF, Lenassi M, Lim SK, Mahoney MG, Mohanty S, Möller A, Nieuwland R, Ochiya T, Sahoo S, Torrecilhas AC, Zheng L, Zijlstra A, Abuelreich S, Bagabas R, Bergese P, Bridges EM, Brucale M, Burger D, Carney RP, Cocucci E, Crescitelli R, Hanser E, Harris AL, Haughey NJ, Hendrix A, Ivanov AR, Jovanovic-Talisman T, Kruh-Garcia NA, Ku’ulei-Lyn Faustino V, Kyburz D, Lässer C, Lennon KM, Lötvall J, Maddox AL, Martens-Uzunova ES, Mizenko RR, Newman LA, Ridolfi A, Rohde E, Rojalin T, Rowland A, Saftics A, Sandau US, Saugstad JA, Shekari F, Swift S, Ter-Ovanesyan D, Tosar JP, Useckaite Z, Valle F, Varga Z, Pol E van der, Herwijnen MJC van, Wauben MHM, Wehman AM, Williams S, Zendrini A, Zimmerman AJ, MISEV Consortium, Théry C, Witwer KW. Minimal information for studies of extracellular vesicles (MISEV2023): From basic to advanced approaches. *J Extracell Vesicles* 2024;**13**:e12404.

10. Balbi C, Bolis S, Vassalli G, Barile L. Flow Cytometric Analysis of Extracellular Vesicles from Cell-conditioned Media. *J Vis Exp* 2019.

11. Rocchetti M, Sala L, Dreizehnter L, Crotti L, Sinnecker D, Mura M, Pane LS, Altomare C, Torre E, Mostacciuolo G, Severi S, Porta A, De Ferrari GM, George AL, Schwartz PJ, Gnecchi M, Moretti A, Zaza A. Elucidating arrhythmogenic mechanisms of long-QT syndrome CALM1-F142L mutation in patient-specific induced pluripotent stem cell-derived cardiomyocytes. *Cardiovasc Res* 2017;**113**:531–541.

12. Richards DJ, Li Y, Kerr CM, Yao J, Beeson GC, Coyle RC, Chen X, Jia J, Damon B, Wilson R, Starr Hazard E, Hardiman G, Menick DR, Beeson CC, Yao H, Ye T, Mei Y. Human cardiac organoids for the modelling of myocardial infarction and drug cardiotoxicity. *Nat Biomed Eng* 2020;**4**:446–462.

13. McNamee N, Catalano M, Mukhopadhya A, O’Driscoll L. An extensive study of potential inhibitors of extracellular vesicles release in triple-negative breast cancer. *BMC Cancer* 2023;**23**:654.

14. Essandoh K, Yang L, Wang X, Huang W, Qin D, Hao J, Wang Y, Zingarelli B, Peng T, Fan G-C. Blockade of exosome generation with GW4869 dampens the sepsis-induced inflammation and cardiac dysfunction. *Biochim Biophys Acta* 2015;**1852**:2362–2371.

15. Tu C, Du Z, Zhang H, Feng Y, Qi Y, Zheng Y, Liu J, Wang J. Endocytic pathway inhibition attenuates extracellular vesicle-induced reduction of chemosensitivity to bortezomib in multiple myeloma cells. *Theranostics* 2021;**11**:2364–2380.

16. Atai NA, Balaj L, Veen H van, Breakefield XO, Jarzyna PA, Van Noorden CJF, Skog J, Maguire CA. Heparin blocks transfer of extracellular vesicles between donor and recipient cells. *J Neurooncol* 2013;**115**:343–351.

17. Hanson B, Vorobieva I, Zheng W, Conceição M, Lomonosova Y, Mäger I, Puri PL, El Andaloussi S, Wood MJA, Roberts TC. EV-mediated promotion of myogenic differentiation is dependent on dose, collection medium, and isolation method. *Mol Ther Nucleic Acids* 2023;**33**:511–528.

**Supplementary Figure**


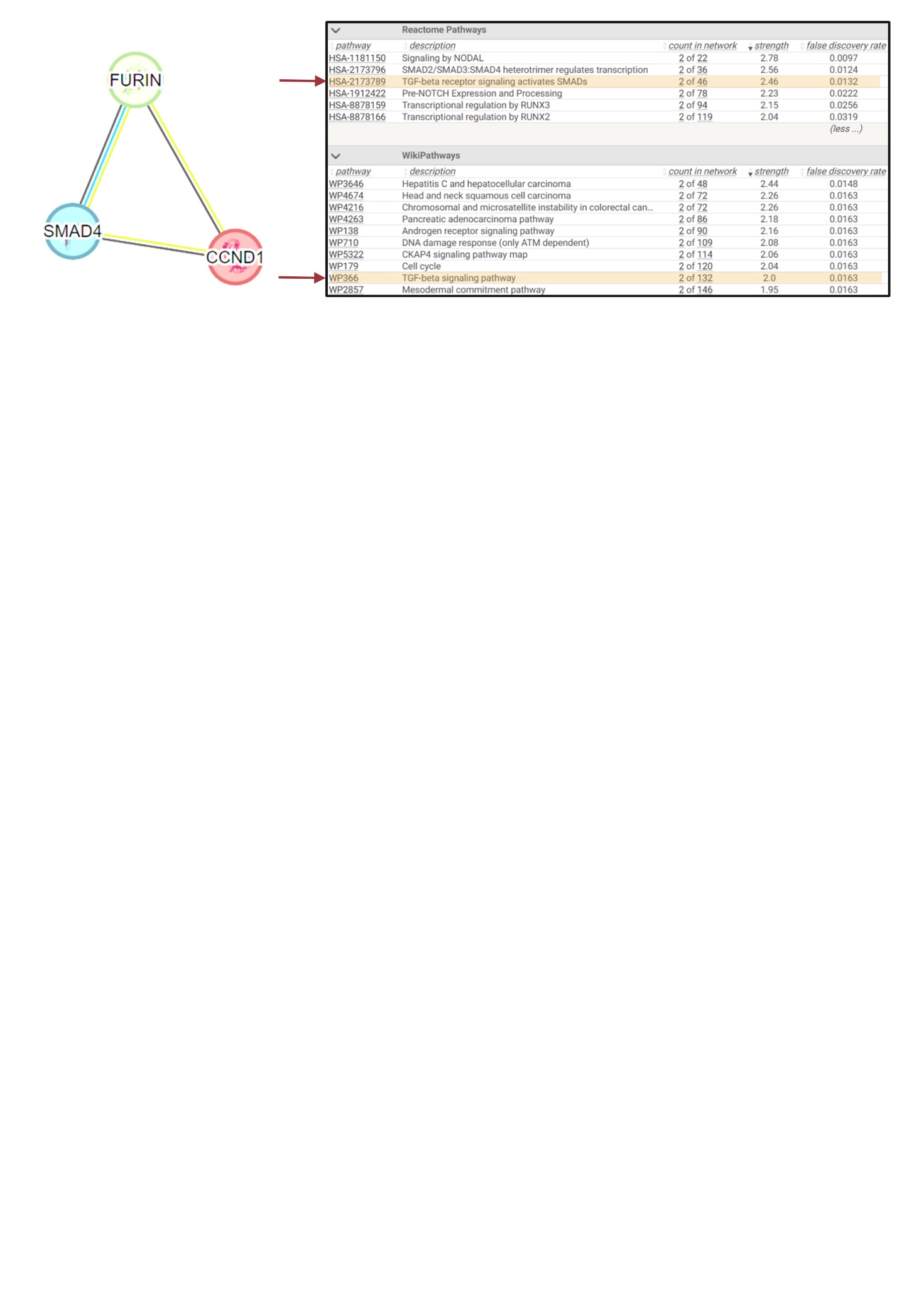


**Supplementary Figure 1**

STRING analysis on FURIN, CCND1 and SMAD4 proteins. The read-out, obtained on Reactome Pathways and WiKiPathways, illustrates that these proteins are involved in the TGFβ signalling pathway.

**
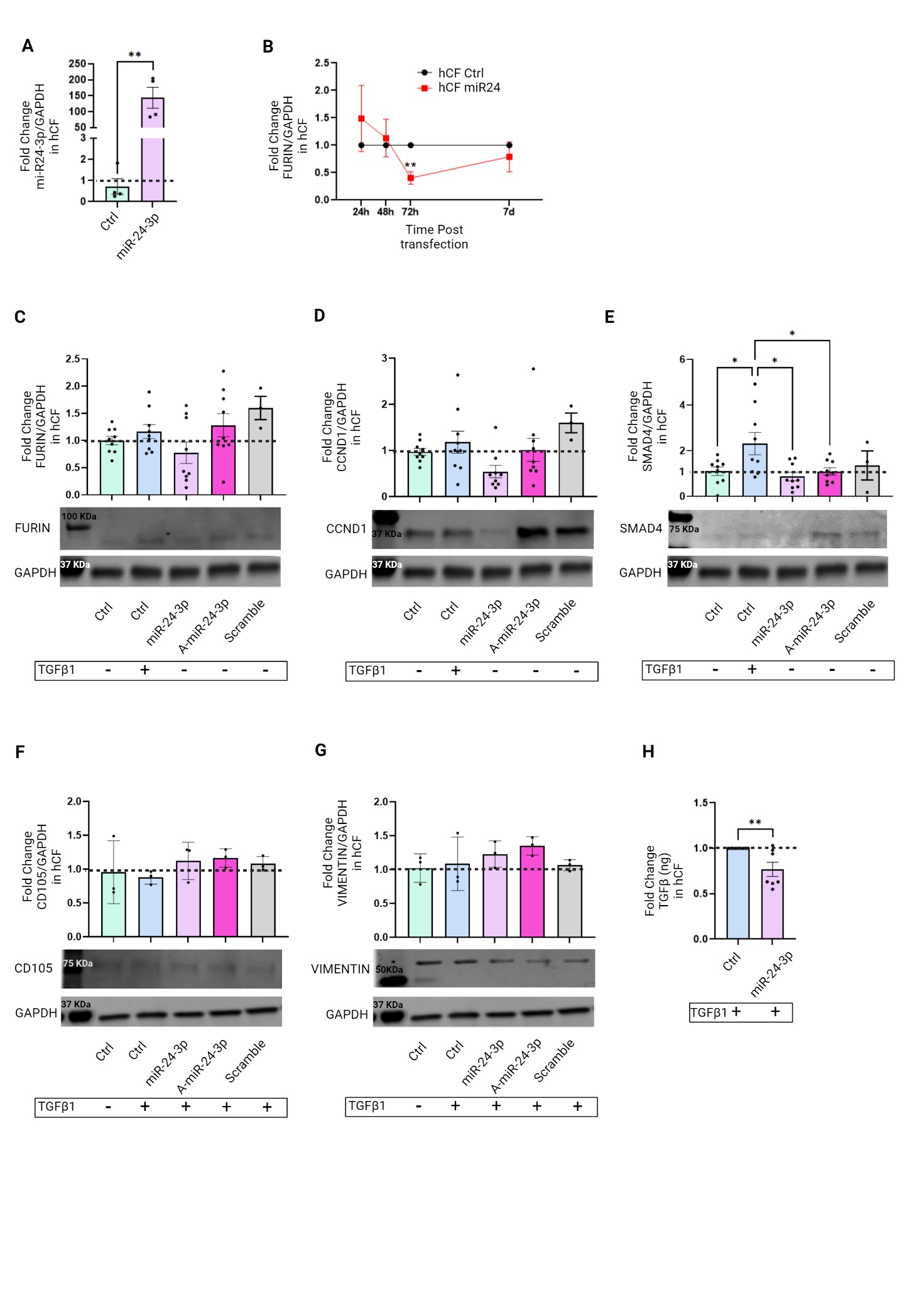
**

**Supplementary Figure 2**

**A)** Real-Time PCR analysis of miR-24-3p expression by naïve hCF (Ctrl) and hCF transfected with a miR-24-3p mimic. **B)** Time-dependent changes in FURIN expression in hCF transfected with a miR-24-3p mimic, as compared with naive hCF (hCF Ctrl). **C)** Western analysis of FURIN expression by either naive or miR-24-3p–transfected hCF at 72 hrs post-transfection. **D)** Western analysis for CCND1 expression*.* **E)** Western analysis for SMAD4 expression. **F)** Western analysis for CD105 expression. **G)** Western analysis for Vimentin expression (data in panels B-G are fold-changes over naïve, untreated hCF; A-miR-24-3p, anti-miR-24-3p and scramble miRNA were used as a control; GAPDH was used as loading control. **H)** TGFβ1 concentrations in conditioned media of TGFβ1-treated, either non-transfected or miR-24-3p–transfected hCF measured by ELISA at 72 hrs post-transfection.


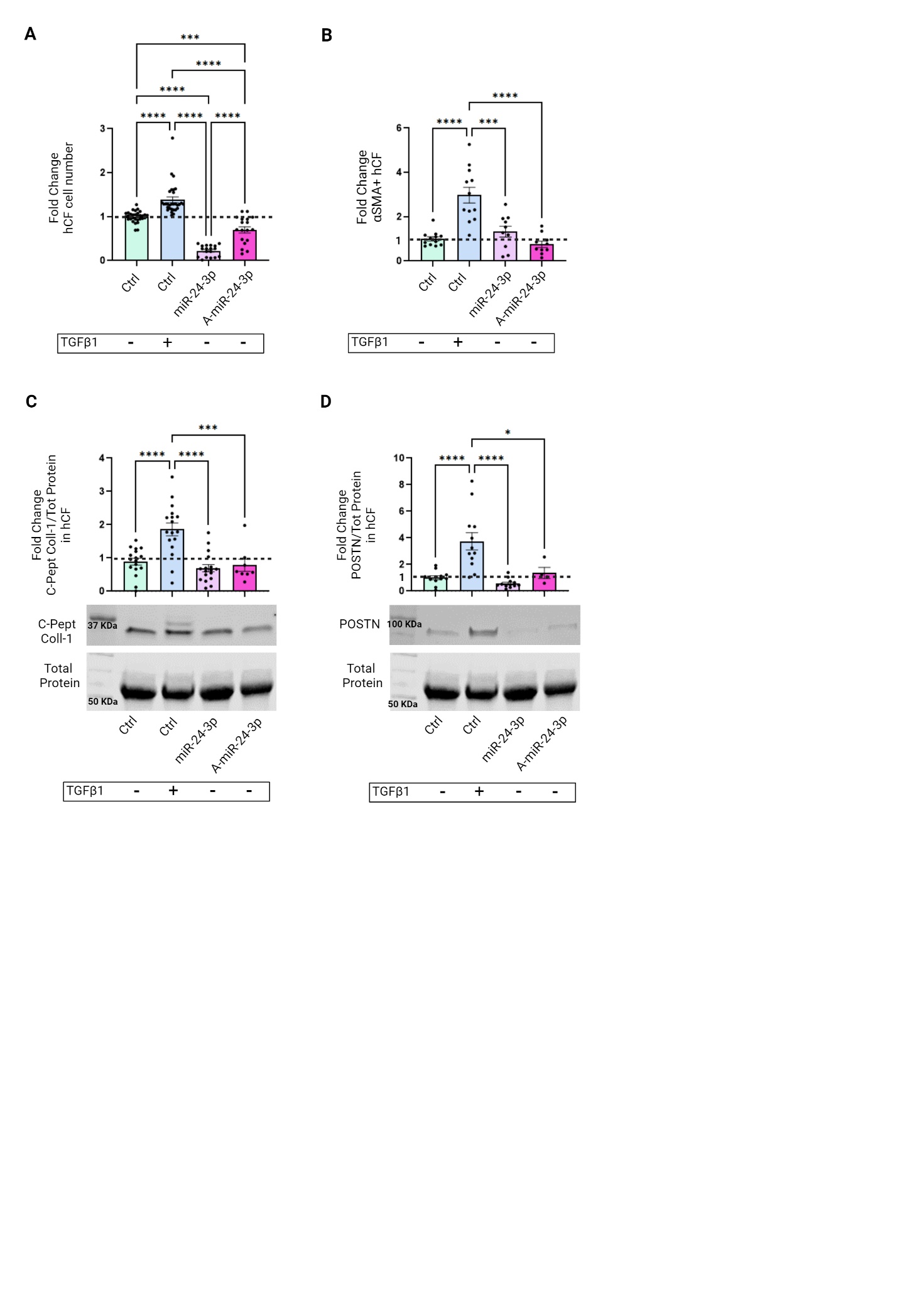


**Supplementary Figure 3**

**A)** hCF numbers at day 7 post-TGFβ1 treatment or transfection with a miR-24-3p mimic. **B)** Quantitative analysis of αSMA^+^ hCF. **C)** Western analysis of collagen-1 C-peptide in hCF conditioned media. **D)** Western analysis of periostin (POSTN) in hCF conditioned media of TGFβ-treated or miR-24-3p transfected hCF (data are fold-changes for the indicated experimental conditions over naïve, untreated hCF). Total protein signal was used as loading control.

**
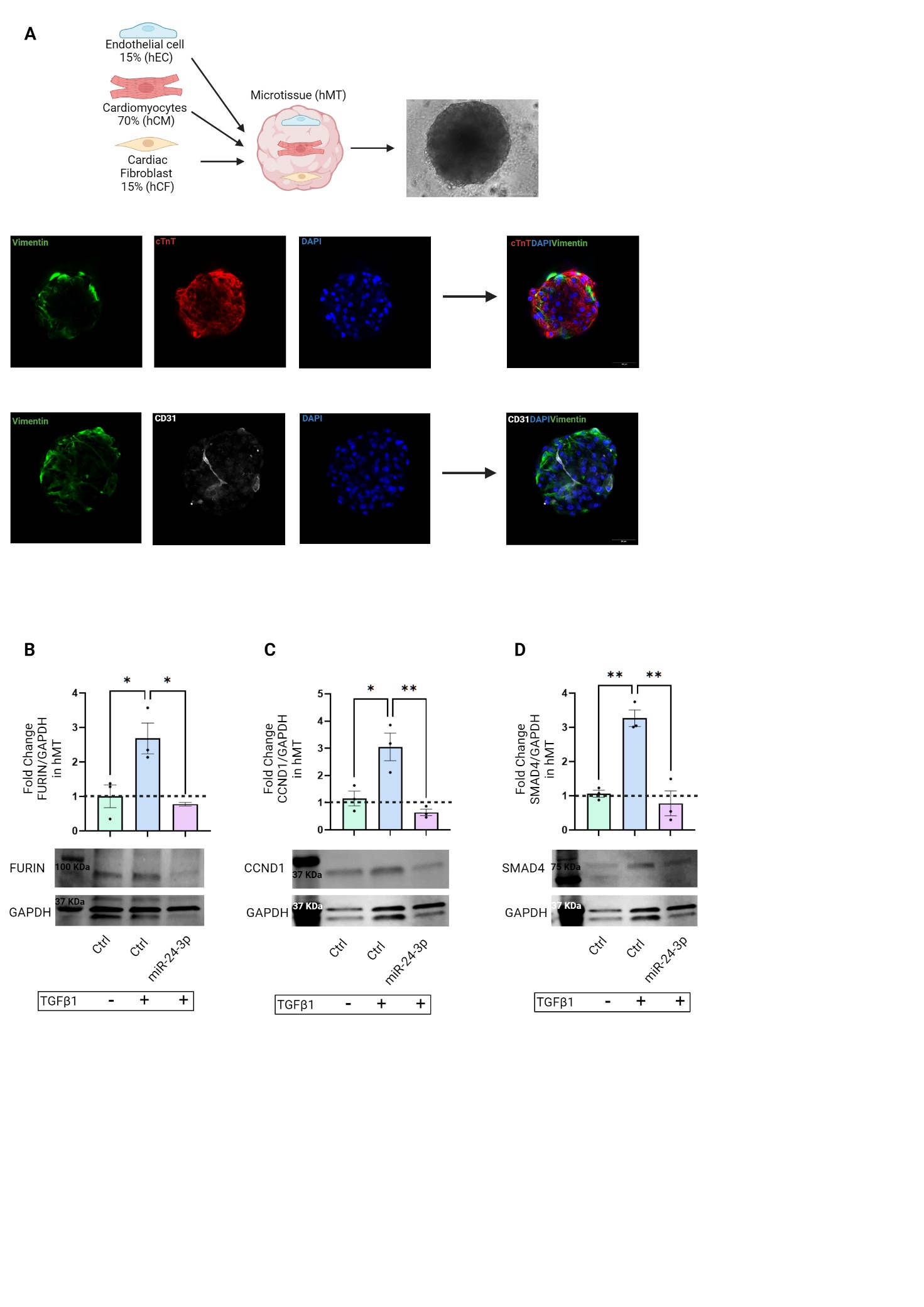
**

**Supplementary Figure 4**

**A)** Schematic figure of the hMT model and representative immunofluorescence images illustrating the 2 cell types present in hMT: Vimentin (hCF), green; cTnT (hCM), red; CD31 (hEC), white; DAPI, blue; scale bar: 50µm. **B)** Western analysis of FURIN expression by TGFβ-treated, naïve or miR-24-3p–transfected hMT at 72 hrs post-transfection. **C)** Western analysis for CCND1 expression*.* **D)** Western analysis for SMAD4 expression (data in panels B-D are fold-changes for experimental conditions over naïve, untreated hCF).

**
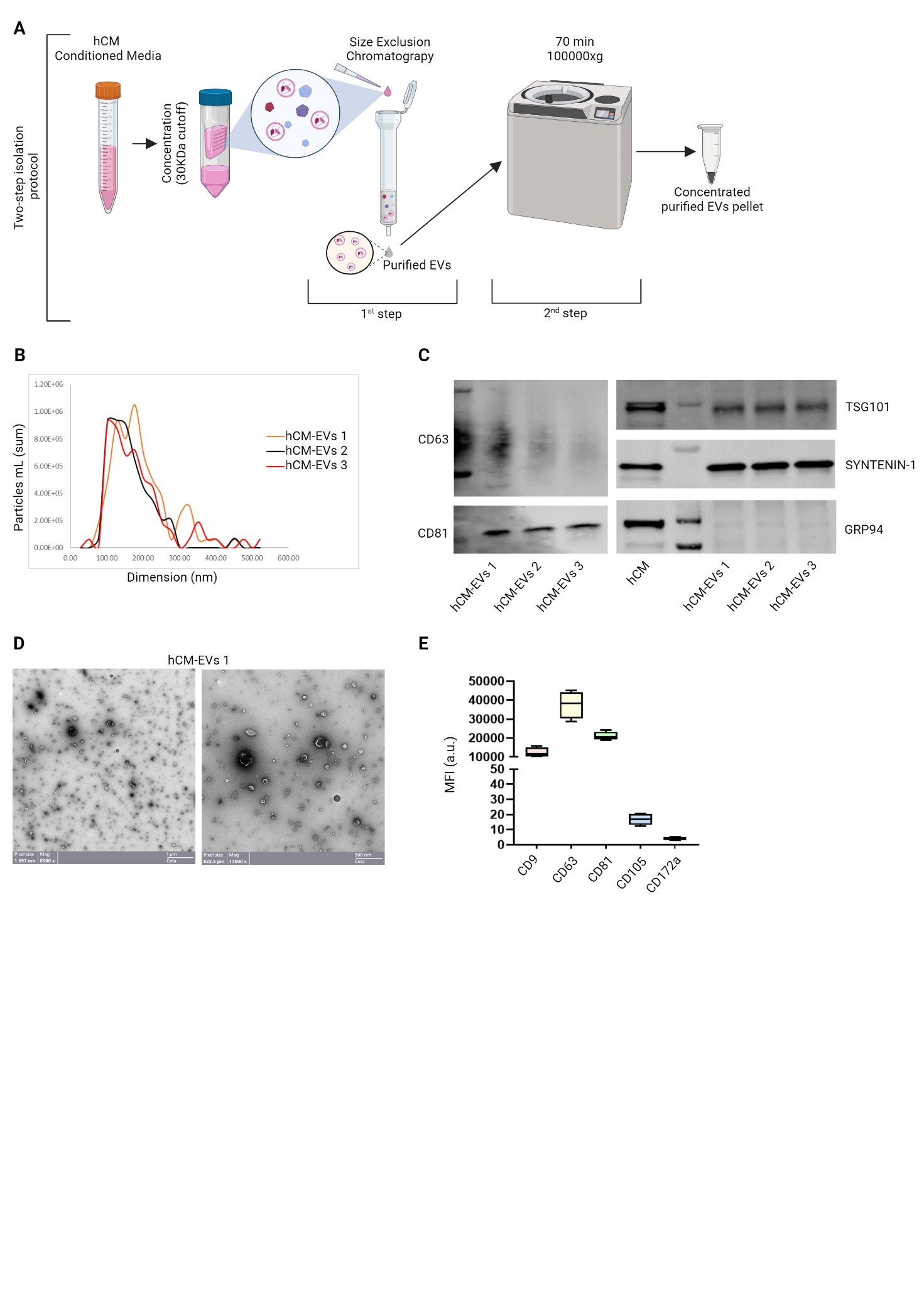
**

**Supplementary Figure 5**

**A)** Schematic representation of EV isolation. **B)** Nanoparticle tracking analysis of 3 different isolation procedures of hCM-EVs. **C)** Western blotting analysis of EVs markers including CD63, CD81, TSG101 and SYNTENIN-1; GRP94 was used as a negative control for contaminating cellular debris. **D)** TEM analysis of hCM-EVs, acquired at 1 µm (left) and 500 nm (right). **E)** Flow cytometry analysis of hCM-EVs for CD9, CD63 and CD81 (EV markers) expression, as well as for CD105 and CD172 expression (iPS-CM markers; MFI, mean fluorescence intensity).

**
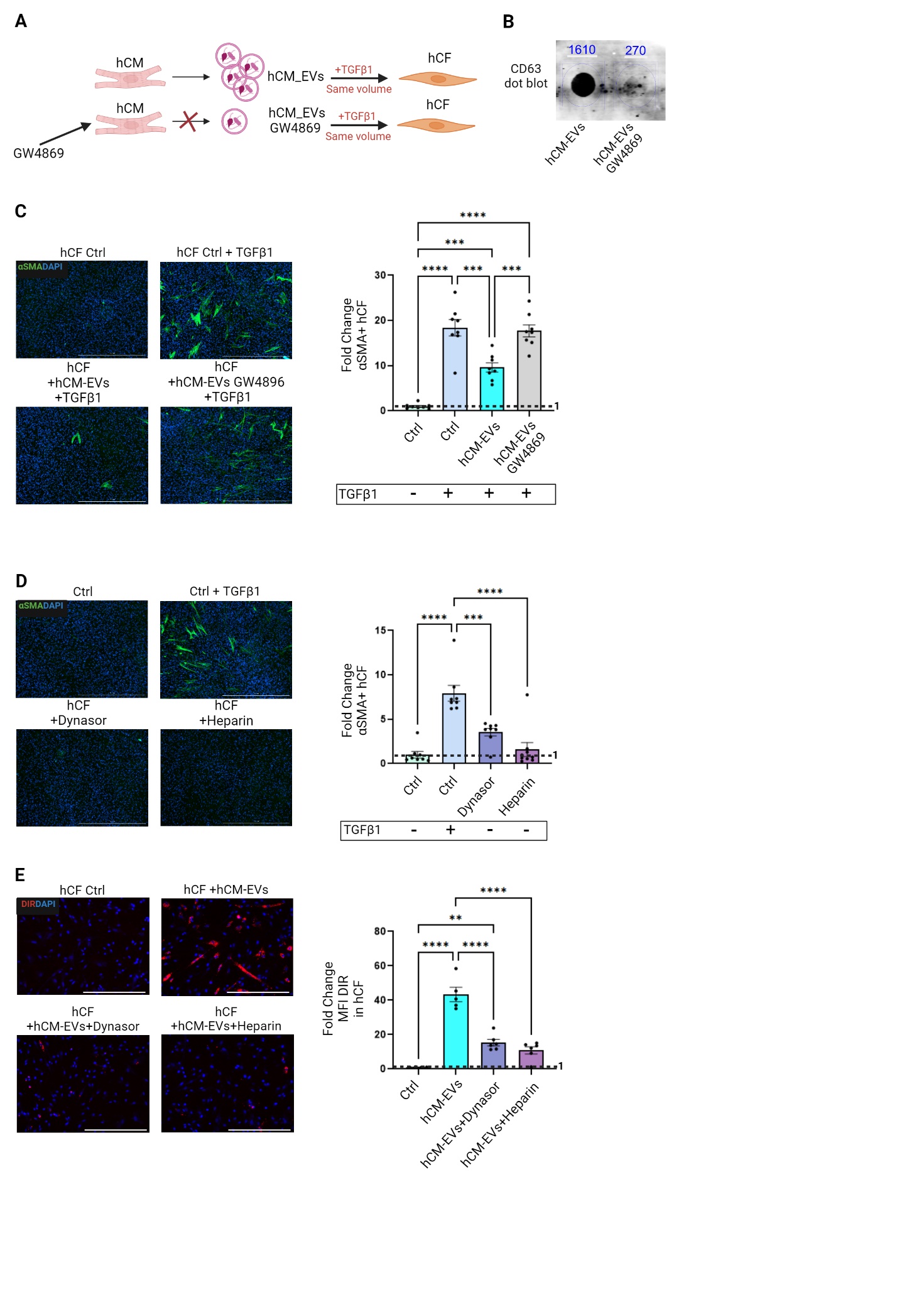
**

**Supplementary Figure 6**

**A)** Schematic representation of hCM-EVs production in the presence or absence of GW4869. **B)** Dot-blot analysis of CD63 expression on same volumes of hCM-EVs and hCM-EVs-GW4869. **C)** Left: Representative immunocytochemical images (αSMA, green; DAPI, blue; scale bar: 1000µm). Right: Quantitative analysis of αSMA^+^ hCF; hCM-EVs reduced αSMA^+^ cell numbers in TGFβ1-treated cells; same volumes of hCM-EVs-GW4869 lacked this effect. **D)** Left: Representative immunocytochemical images (αSMA, green; DAPI, blue; scale bar: 1000µm). Right: Quantitative analysis of αSMA^+^ hCF. Dynasor- or heparin-treated cells did not differ from untreated cells. **E)** Left: Representative immunocytochemical images (DiR, red; DAPI, blue; scale bar: 1000µm). Right: Quantitative analysis of DiR fluorescence, indicating hCM-EVs uptake (data are fold-changes for the indicated experimental conditions over naive hCF).

**
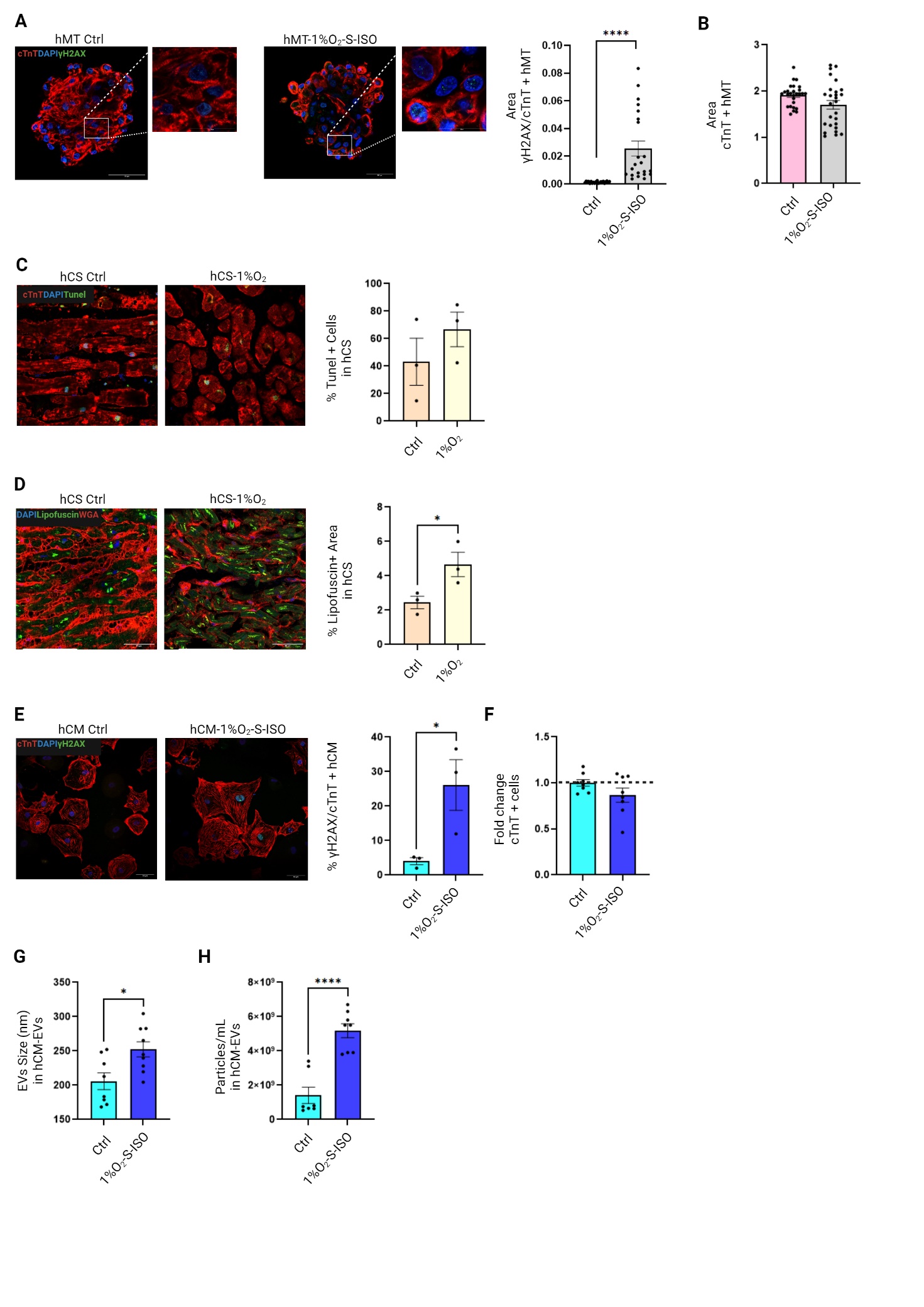
**

**Supplementary Figure 7:**

**A)** Left panel: Representative immunofluorescence images (cTnT in red, ƴH2AX in green, DAPI in blue; scale bar 50 µm); Right panel: Quantitative analysis of ƴH2AX/cTnT^+^ cell area on hMT after 7 days of ischaemia-simulating conditions [i.e., hypoxia (1% O_2_) combined with serum starvation (S) and 500 µM isoproterenol (ISO); (hMT-1%O_2_-S-ISO)]. **B)** Quantitative analysis of cTnT^+^ cell area on hMT after 7 days after ischaemia- simulating conditions. **C)** Left panel: Representative immunofluorescence images (cTnT in red, TUNEL in green, DAPI in blue; scale bar 50 µm). Right panel: Quantitative analysis of % TUNEL^+^ cells on hCS cultured for 7 days under either hypoxic (1% O_2_) or normoxic conditions*.* **D)** Left panel: Representative immunofluorescence images (WGA in red, Lipofuscin in green, DAPI in blue; scale bar 50 µm). Right panel: Quantitative analysis of Lipofuscin^+^ area on hCS cultured for 7 days under either hypoxic or normoxic conditions*.* **E)** Left panel: Representative immunofluorescence images (cTnT in red, ƴH2AX in green, DAPI in blue; scale bar 50 µm). Right panel: Quantitative analysis of ƴH2AX^+^/cTnT^+^ on hCM cultured under ischaemia-simulating conditions*.* **F)** Fold changes of cTnT^+^ cells on hCM cultured under ischaemia-simulating conditions*.* **G)** Size dimension analysis of hAC-EVs under normoxic or ischaemia-simulating conditions. **H)** Particle number analysis of hAC-EVs under such conditions*.*

**
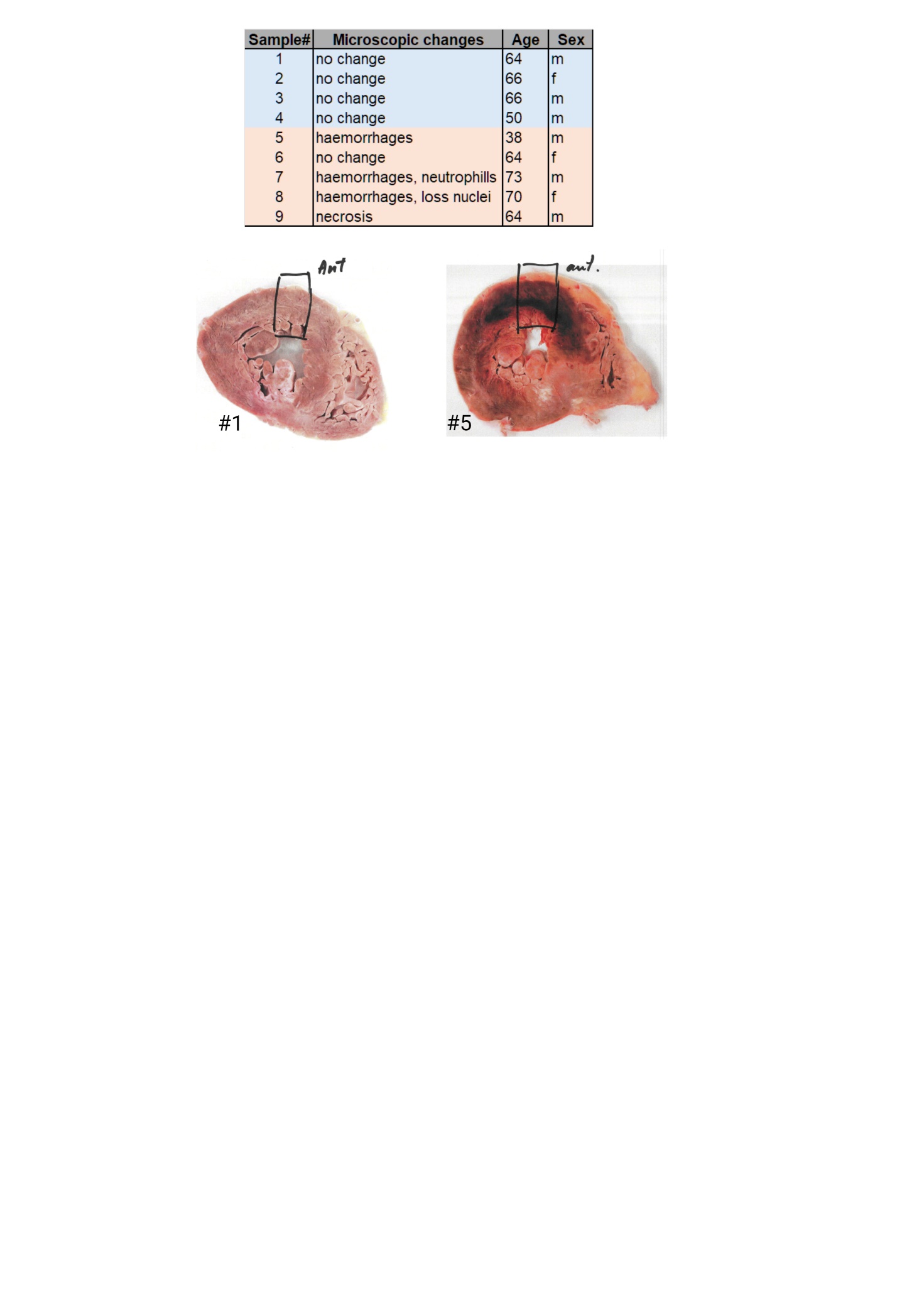
**

**Supplementary Figure 8:** Post-mortem human heart samples from donors who died from either disease conditions not associated with heart disease (n=4) or from acute myocardial infarction (n=5).


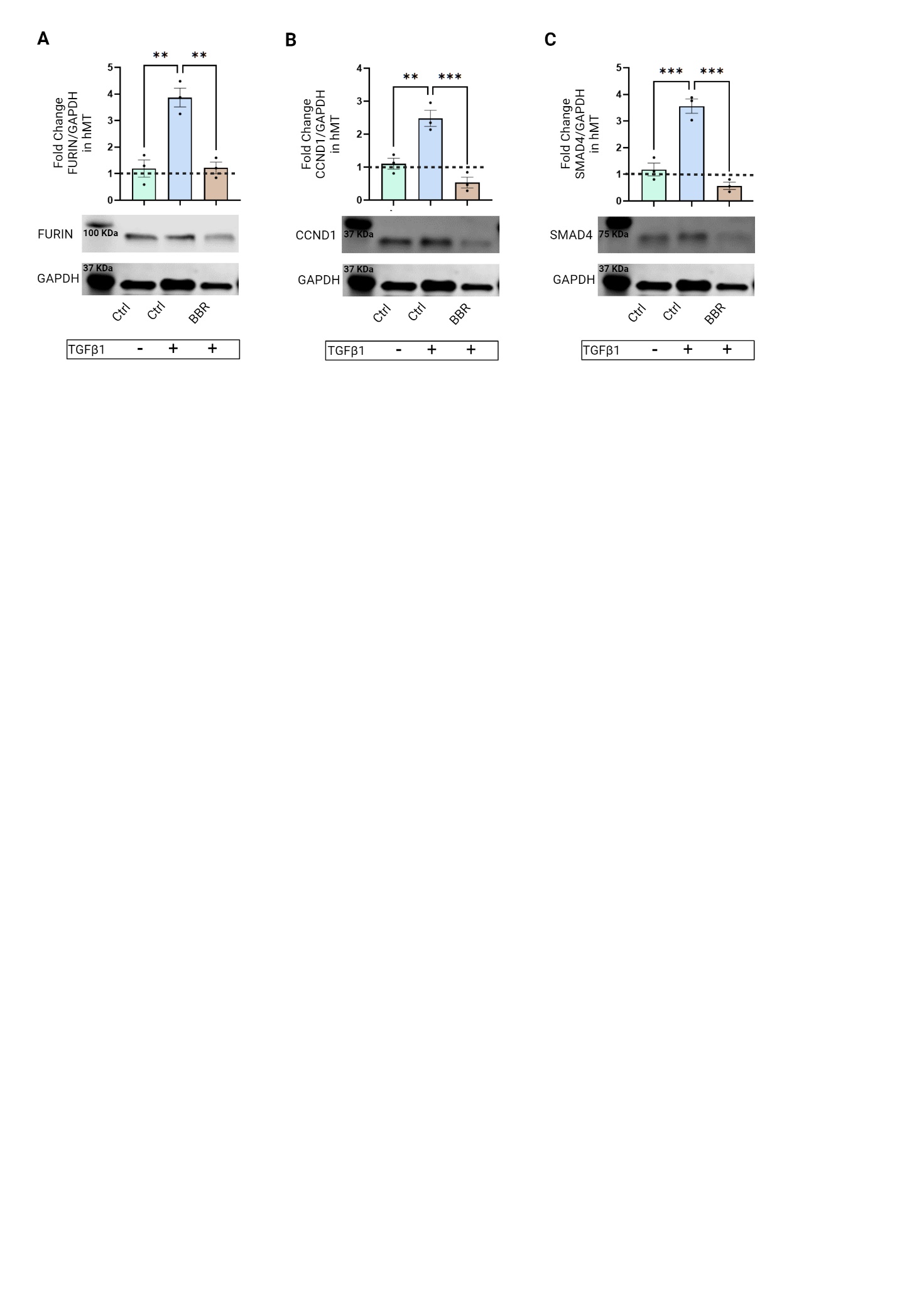


**Supplementary Figure 9:**

**A)** Western analysis of FURIN expression by TGFβ1-treated and/or BBR-treated hMT at 72 hrs. **B)** Western analysis for CCND1 expression. **C)** Western analysis for SMAD4 expression (data are fold-changes for treated hCF over untreated ones).
